# The CAGE–MiR-181b-5p–S1PR1 Axis Regulates Anticancer Drug Resistance and Autophagy in Gastric Cancer Cells

**DOI:** 10.3389/fcell.2021.666387

**Published:** 2021-05-25

**Authors:** Minjeong Yeon, Youngmi Kim, Deepak Pathak, Eunju Kwon, Dong Young Kim, Myeong Seon Jeong, Hyun Suk Jung, Dooil Jeoung

**Affiliations:** ^1^Department of Biochemistry, College of Natural Sciences, Kangwon National University, Chuncheon, South Korea; ^2^Institute of New Frontier Research, College of Medicine, Hallym University, Chuncheon, South Korea; ^3^College of Pharmacy, Yeungnam University, Gyeongsan, South Korea; ^4^Chuncheon Center, Korea Basic Science Institute, Chuncheon, South Korea

**Keywords:** anticancer drug resistance, autophagic flux, cancer-associated gene, miR-181b-5p, sphingosine 1-phosphate receptor

## Abstract

Cancer-associated gene (CAGE), a cancer/testis antigen, has been known to promote anticancer drug resistance. Since the underlying mechanisms of CAGE-promoted anticancer drug resistance are poorly understood, we established Anticancer drug-resistant gastric cancer cells (AGS^*R*^) to better elucidate possible mechanisms. AGS^*R*^ showed an increased expression level of CAGE and autophagic flux compared with anticancer drug-sensitive parental gastric cancer cells (AGS cells). AGS^*R*^ cells showed higher invasion potential, growth rate, tumor spheroid formation, and angiogenic potential than AGS cells. CAGE exerted effects on the response to anticancer drugs and autophagic flux. CAGE was shown to bind to Beclin1, a mediator of autophagy. Overexpression of CAGE increased autophagic flux and invasion potential but inhibited the cleavage of PARP in response to anticancer drugs in CAGE CRISPR–Cas9 cell lines. TargetScan analysis was utilized to predict the binding of miR-302b-5p to the promoter sequences of CAGE, and the results show that miR-302b-5p directly regulated CAGE expression as illustrated by luciferase activity. MiR-302b-5p regulated autophagic flux and the response to anticancer drugs. CAGE was shown to bind the promoter sequences of miR-302b-5p. The culture medium of AGS^*R*^ cells increased CAGE expression and autophagic flux in AGS cells. ImmunoEM showed CAGE was present in the exosomes of AGS^*R*^ cells; exosomes of AGS^*R*^ cells and human recombinant CAGE protein increased CAGE expression, autophagic flux, and resistance to anticancer drugs in AGS cells. MicroRNA array revealed miR-181b-5p as a potential negative regulator of CAGE. MiR-181b-5p inhibitor increased the expression of CAGE and autophagic flux in addition to preventing anticancer drugs from cleaving poly(ADP-ribose) polymerase (PARP) in AGS cells. TargetScan analysis predicted sphingosine 1-phosphate receptor 1 (SIPR1) as a potential target for miR-181b-5p. CAGE showed binding to the promoter sequences of S1PR1. The downregulation or inhibition of S1PR1 led to decreased autophagic flux but enhanced the sensitivity to anticancer drugs in AGS^*R*^ cells. This study presents a novel role of the CAGE–miR-181b-5p–S1PR1 axis in anticancer drug resistance and autophagy.

## Introduction

Cancer-associated gene (CAGE), a cancer/testis gene, was initially discovered in the sera of patients with gastric cancers (Cho et al., 2002). The expression level of CAGE was closely related to the methylation status of CAGE promoter sequences (Cho et al., 2003). Anti-CAGE antibody was shown to be present in 7 of 13 (53.8%) patients with microsatellite instability-positive endometrial cancer and in 1 of 3 patients with atypical endometrial hyperplasia (Iwata et al., 2005). This suggests that CAGE could be utilized as a marker for the detection of microsatellite instability-positive endometrial cancers. There was the presence of CAGE in the sera of 12% of early-stage gastric cancer patients (Hurtado López et al., 2020). In addition, CAGE shows promise as an immunotherapeutic target in chronic myelomonocytic leukemia (Hurtado López et al., 2020).

Cancer-associated gene also enhances cellular proliferation by increasing the expression of cyclinD1 in an AP-1-dependent manner (Por et al., 2010). CAGE induces anticancer drug resistance by decreasing the expression of p53 (Kim et al., 2010). MiR-200b enhances the sensitivity of anticancer drugs by decreasing the expression of CAGE (Kim et al., 2013). CAGE is crucial to confer resistance to Taxol in cervical cancer cells (Park et al., 2018). CAGE increases cyclin D1 expression and enhances resistance to anticancer drugs through binding to glycogen synthase kinase 3 β (GSK3β) (Kim et al., 2017a). CAGE enhances the self-renewal and tumorigenic potential of anticancer drug-sensitive melanoma cells (Kim et al., 2017b).

Autophagy is closely associated with the response to anticancer drugs in many types of cancer. UTI (ulinastatin) inhibits EPI (epirubicin)-induced autophagy, promotes apoptosis, and enhances sensitivity to EPI in hepatic cancer cells (Song et al., 2015). Autophagy inhibition sensitizes breast cancer cells to paclitaxel (Wang et al., 2017). Autophagy is induced by chemotherapy and is associated with chemoresistance (Belounis et al., 2016). The inhibition of autophagy significantly enhanced AGEs (advanced glycation end products) and induced cell apoptosis (Xu et al., 2016). Thus, CAGE may play a role in autophagy.

Cancer-associated gene binds to epidermal growth factor receptor (EGFR) and increases self-renewal, in addition to increasing autophagic flux (Kim et al., 2016). The CAGE–miR-140-5p–proto-oncogene Wnt1 axis regulates autophagic flux in colon cancer cells (Yeon et al., 2019). CAGE promotes cellular interactions mediated by exosomes (Yeon et al., 2019). These reports suggest that CAGE may regulate anticancer drug resistance and autophagic flux by mediating cellular interactions.

MiR-300 activates protein phosphatase 2A (PP2A) and is necessary for anticancer drug resistance conferred by leukemic stem cells (Silvestri et al., 2020). *TUG1* long noncoding RNA is necessary for the survival of these leukemic stem cells by regulating the apoptotic function of miR-300 function (Silvestri et al., 2020). MiR-200b negatively regulates CAGE expression and enhances sensitivity to anticancer drugs in melanoma cells (Kim et al., 2013). MiR-217 enhances anticancer drug sensitivity by regulating CAGE expression and the interaction between CAGE and EGFR (Kim et al., 2016). These reports imply the roles of miRNAs in anticancer drug resistance and autophagy.

In this study, we found that both anticancer drug resistance and autophagic flux were regulated by a CAGE–miR-302b-5p negative feedback loop and displayed a close relationship. We showed that CAGE regulated anticancer drug resistance and was present in the exosomes of anticancer drug-resistant gastric cancer cells (AGS^*R*^). The miR-181b-5p–S1PR1 axis acts as a negative regulator of anticancer drug resistance and autophagic flux. CAGE was shown to act as a direct regulator of sphingosine 1-phosphate receptor 1 (S1PR1) expression. Taking our data as a whole, we clearly demonstrate that the CAGE–miR-181b-5p–S1PR1 axis can be utilized as a target for the development of anticancer therapeutics.

## Materials and Methods

### Materials

We purchased chemicals from Sigma Chemical Company. We purchased anti-mouse and anti-rabbit IgG-horseradish peroxidase conjugate antibody from Pierce Company (Rockford, IL, United States). Lipofectamine and PLUS^TM^ reagent for transfection were purchased from Invitrogen (San Diego, CA, United States). Oligonucleotides, miRNA mimic, miR inhibitors, and siRNAs used in this study were purchased from Bioneer Company (Daejeon, Korea). We purchased tissue microarray from Signosis (California, United States).

### Cell Lines and Cell Culture

Cancer cell lines were cultured in Dulbecco’s modified minimal essential medium (Invitrogen) supplemented with heat-inactivated 10% fetal bovine serum (Invitrogen) and antibiotics. Cells were maintained at 37°C in 5% CO_2_. Anticancer drug-resistant cancer cell lines were established as described (Kim et al., 2010).

### Generation of Knockout Cell Line With the CRISPR/Cas9 System

To generate CAGE-deficient AGS^*R*^ cells, CRISPR/Cas9-mediated gene editing was performed. A plasmid encoding Cas9 was purchased from ToolGen. For sgRNA expression, the hU6-sgRNA plasmid that targeted CAGE (5′-AGGCTAATCCAAGAGACCTTGGG-3′) was used (ToolGen). AGS^*R*^ cells were transfected with Cas9, hU6-sgRNA, and hygromycin B-resistant reporter plasmid (ToolGen). After 48 h of transfection, cells were treated with hygromycin B (150 μg/ml) three times a week. Hygromycin-resistant colonies were isolated and subjected to immunoblot.

### Colony Formation

Colonies were stained with 0.01% crystal violet and counted.

### Cell Viability Determination

MTT assays were employed to determine the response to anticancer drugs. Viable cell number counting was carried out by trypan blue exclusion assays.

### Matrigel Plug Assays

BALB/C mice (Nara Biotech) were given a subcutaneous injection with 0.1 ml of Matrigel containing culture medium and 10 units of heparin (Sigma). Hemoglobin (Hb) content in the Matrigel plugs was measured using Drabkin’s reagent (Sigma, United States).

### Chemo Invasion Assays

A transwell chamber system with 8-μm pore polycarbonate filter inserts (CoSTAR, Acton, MA, United States) was employed. Trypsinized cells (5 × 10^3^) in the serum-free RPMI 1,640 medium containing 0.1% bovine serum albumin were added to each upper chamber of the transwell. RPMI 1,640 medium supplemented with 10% fetal bovine serum was placed in the lower chamber and cells were incubated at 37°C for 16 h. The invaded cells were stained and counted as described (Kim et al., 2017a). Differences were considered significant when *p* < 0.05.

### Tumor Spheroid-Forming Potential

Cells were plated (5 × 10^4^ cells/well) in ultralow attachment plates (Corning Inc.) in DMEM/F12 stem cell medium. Cells were fed with 0.2 ml of fresh stem cell medium on days 2, 4, and 6. The total number of spheres was counted after 7 days by inverted microscopy (Olympus).

### RNA Extraction and Quantitative Real-Time PCR

Total miRNA was isolated using the *mir*Vana miRNA isolation kit (Ambion) and was extended by a poly(A) tailing reaction using the A-Plus poly(A) polymerase tailing kit (CellScript). CDNA was synthesized according to the manual provided by the manufacturer (Quanta Biogenesis). Levels of miRNAs were determined by a SYBR Green qRT-PCR kit (Ambion). The expression of miR-302b-5p was defined based on the threshold (*C*_*t*_), and relative expression levels were determined as 2^–((^*^*Ct*^* ^*of miR 302b–5p*)^^–^^(^*^*Ct*^* ^*of U6))*^ after normalization to the expression of U6 small nuclear RNA. Primer sequences are listed in the Supplementary Tables.

### MiRNA Target Analysis

Genes that contain the miRNA-binding site(s) in the UTR were obtained using the http://TargetScan program^1^, Diana laboratory^2^, and miRDB^3^.

### Transfection

Cells were transiently transfected with the miRNA inhibitor, miRNA mimic, or siRNA (each at 10 nM) with jetPRIME^®^ (Polyplus, cat. 114–15). The sequences of miR mimic, miR inhibitors, and siRNAs are listed in the Supplementary Tables.

### Luciferase Activity Assays

PCR-amplified 3′ UTR of S1PR1 (381 bp) was cloned into the *Xba*I site of pGL3 luciferase plasmid. The mutant pGL3–3′ UTR–S1PR1 construct was made with the QuikChange site-directed mutagenesis kit (Stratagene). Luciferase activity assay was performed as described (Kim et al., 2019).

### Immunofluorescence Staining

Cells were washed and fixed with 4% paraformaldehyde before permeabilization with Triton X-100. After blocked with goat serum (10%) in 0.1% BSA/PBS, cells were incubated with anti-LC3 or anti-CAGE at 4°C overnight and then incubated with anti-rabbit Alexa Fluor 488 (for LC3 and CAGE) secondary antibody. After removal of antibodies, cells were stained with DAPI and mounted with mounting medium. The immune fluorescent images were observed and captured using a confocal laser scanning microscope.

### Immunoblot and Immunoprecipitation

Immunoblot and immunoprecipitation were performed as described (Yeon et al., 2019). Cell lysates were prepared using lysis buffer [62.5 mM Tris–HCl, pH 6.8, 2% (w/v) SDS, 10% (v/v) glycerol, 50 mM dithiothreitol, 0.01% (w/v) bromphenol blue, 10 mM NaF, 1% (v/v) protease inhibitor mixture (Roche), 1 mM sodium orthovanadate]. The denatured cell lysates (20 μg/well) were analyzed on a 10% SDS-PAGE and were transferred onto PVDF membrane and subjected to immunoblotting. The following primary antibodies were used in this study: CAGE (MBS2524843, MyBioSource); AMPKα (AF3194, R&D Systems); pAMPKα^*Thr172*^ (2535S, Cell Signaling), PARP (9542S, Cell Signaling), pBeclin1^*Ser15*^ (84966S, Cell Signaling), LC3 (12741S, Cell Signaling), Bcl-2 (3498S, Cell Signaling), E-cadherin (3195S, Cell Signaling), vimentin (5741S, Cell Signaling), mTOR (2972S, Cell Signaling), pmTOR^*Ser*2448^ (2971S, Cell Signaling), Alix (2171S, Cell Signaling), and p53 (2524S, Cell Signaling); Beclin1 (sc-48341, Santa Cruz), IgG (sc-2025, Santa Cruz), SNAIL (sc-271977, Santa Cruz), ATG5 (sc-133158, Santa Cruz), PAI-1 (sc-5297, Santa Cruz), biotin (sc-101339, Santa Cruz), TSG101 (sc-7964, Santa Cruz), FAK (sc-558, Santa Cruz), and CD81 (sc-166029, Santa Cruz); actin (A2228, Sigma) and FLAG (F3166, Sigma); caspase-3 (PA05689A0Rb, Cusabio); p62 (ab56416, Abcam); and S1PR1 (55133-I-AP, Proteintech).

The following secondary antibodies were used in this study: anti-mouse HRP secondary antibody (31430, Invitrogen), anti-goat HRP secondary antibody (31402, Invitrogen), anti-rabbit HRP secondary antibody (ADI-SAB-300-J, Enzo), and anti-rabbit Alexa Fluor 488 secondary antibody (A11008, Invitrogen).

### Chromatin Immunoprecipitation Assays

Assays were performed using a kit from Upstate Company. For detection of the binding of CAGE to miR-302b-5p promoter sequences, specific primers of miR-302b-5p promoter-1 sequences [5′-TCTGTTTCATTTCTGACTCT-3′ (sense) and 5′-CCAAGTCATTGTGAATGTAT-3′ (antisense)], miR-302b-5p promoter-2 sequences [5′-GCCAATTAAATTTTTGAGTGT CTG-3′ (sense) and 5′-ACGGGGTGTTTTGTTCTACT-3′ (antisense)], and miR-302b-5p promoter-3 sequences [5′-CCACCCAGGATCATACATTC-3′ (sense) and 5′-AAAGATTC GTGTTCTCCTCC-3′ (antisense)] were used. For binding of CAGE to S1PR1 promoter sequences, specific primers of S1PR1 promoter-1 sequences [5′-TGGCGGGGGGAG TACAGGAA-3′ (sense) and 5′-TCAGCACACCGATCCTCC TAGGG-3′ (antisense)], S1PR1 promoter-2 sequences [5′-GGCCGTCCTCTGCCTCCTC-3′ (sense) and 5′-TTTGTTG TTTGGGGAGGAGGGGT-3′ (antisense)], and S1PR1 promoter-3 sequences [5′-GCTTCTGCCCCAGATCTTTC CTGG-3′ (sense) and 5′-GGCCATTGGAGTGCTCCGC-3′ (antisense)] were used.

### Electron Microscopic Observation of the Autophagic Process

Cells were treated with the fixation solution [2.5% glutaraldehyde in 0.1 M cacodylate solution (pH 7.0) for 1 h] and then mixed with 2% osmium tetroxide for 2 h at 4°C. The samples were dehydrated with a graded acetone series and embedded into a Spurr medium (Electron Microscopy System). The samples were sectioned (60 nm) by using an ultra-microtome (RMC MTXL, Arizona, United States). The section was stained with 2% uranyl acetate (for 20 min) followed by staining of lead citrate (for 10 min). The sections were viewed under a transmission electron microscope (JEM-2100F, Japan) at 200 kV.

### Isolation of Exosomes

Cells were cultured under serum-free medium (Invitrogen, Carlsbad, CA, United States) and the culture medium was harvested after 48 h of incubation. Isolation of exosomes was carried out by using ExoQuick-TC reagent (System Biosciences, Mountain View, CA, United States). Exosomes were observed under a transmission electron microscope (JEM-2100F, Japan) at 200 kV.

### Size Distribution Analysis of Exosomes

Exosomes were incubated on the ExoView chip (ExoView, United States) for 16 h. The chips were then washed three times in PBS with 0.05% Tween-20 (PBST) and then imaged with the ExoView R100 reader (ExoView, United States) using the ExoScan (ExoView, United States) acquisition software.

### Internalization of Exosomes

Exosomes prepared from AGS^*R*^ cells were labeled with PKH67 Fluorescent Cell Linker kits (Sigma-Aldrich, St. Louis, MO, United States). In order to examine the internalization of exosomes, AGS cells were plated out onto a coverslip (2 × 10^4^ cells). The following day, each medium containing PKH67-labeled exosomes or PKH67-unlabeled exosomes was added into each well for 24 h. After incubation, the coverslips were washed with PBS, and 4% paraformaldehyde solution was then added to the slides and incubated for 15 min. Cells were visualized under a confocal laser scanning microscope LX70 FV300 05-LPG-193 (Olympus, Japan).

### The Presence of CAGE in the Exosomes

Exosomes extracted from AGS^*R*^ cells (REF, KIT model) were subjected to centrifugation at 60,000 × *g* for 30 min to precipitate extracellular vesicles. Collected vesicles were prefixed with 0.1% glutaraldehyde and 2% paraformaldehyde in phosphate buffer (pH 7.4) for 1 h at 4°C and then postfixed in 2% osmium tetroxide for 30 min at 4°C. Samples were dehydrated with a graded series of ethanol and then treated with graded propylene oxide series. Dehydrated samples were embedded into epoxy resin (PELCO, United States). Preparation of ultrathin sections (∼80 nm) was carried out with Ultracut UCT (Leica, Germany). Sections were mounted on copper grids and stained with 1% uranyl acetate and lead citrate (for 10 min) for the subsequent observations. For immunogold labeling electron microscopy, sections (∼80 nm) on the grids were treated with 0.02 M glycine for 10 min. Sections were then washed in deionized water, floated for 1 h in PBS containing 1% BSA, and incubated with the primary rabbit or mouse antibody (anti-CAGE or/and anti-TSG101 antibodies, respectively) at 1:20 dilutions overnight at 4°C. The grids were washed with 0.1% BSA in PBS (five times) and incubated in secondary antibody, anti-rabbit IgG conjugated to 10 nm or anti-mouse IgG conjugated to 25 nm (AURION, Holland) diluted 1:20 in 0.1% BSA–PBS. The sample grids were stained with uranyl acetate and lead citrate. The sectioned and immunogold-labeled grids were examined using a JEOL-2100F transmission electron microscope (JEOL, United States) operated at 200 kV.

### Expression and Purification of CAGE Protein

Full-length CAGE gene (residues 1–631) was inserted into pETDuet-1 vector (Merck Millipore, Billerica, MA, United States) expressing N-terminal 6_*X*_His and thioredoxin followed by TEV protease cleavage site, and then the plasmid was transformed into *Escherichia coli* strain BL21 Star (DE3) (Thermo Fischer Scientific, United States). Transformed cells were grown in LB media at 37°C. The media were cooled when OD_600_ (optical density at 600 nm) reached 0.6∼0.7 and 0.4 mM IPTG was then added into the culture media to induce CAGE expression. After overnight incubation at 15°C, the cells were harvested by using centrifugation at 3,000 × *g* (for 10 min). The cells were resuspended in buffer A (20 mM HEPES pH 7.5, 0.5 m NaCl, 0.2 mM TCEP, and 5% glycerol), lysed by sonication, and clarified by centrifugation at 20,000 × *g* for 30 min after the addition of DNase I and RNase A. CAGE was then purified by IMAC (immobilized metal affinity chromatography) and SEC (size exclusion chromatography). The clarified cell lysate was loaded onto a 5-ml HisTrap nickel chelating column (GE Healthcare Bio-Sciences, Uppsala, Sweden), and the resin was washed with buffer A containing 40 mM imidazole. Proteins bound to the resin were eluted by an imidazole gradient. Fractions that contain CAGE were pooled and treated with TEV protease overnight at 4°C. After complete cleavage, the protein solution was dialyzed against buffer A and passed through the Ni-NTA resin (Thermo Scientific, United States). CAGE was further purified by SEC using Superdex 200 preparatory grade column (GE Healthcare Biosciences, United States) pre-equilibrated with buffer A.

### Tissue Microarray and Immunohistochemical Staining

Immunohistochemical staining of tissue microarray was performed using an avidin–biotin detection method (Vectastain ABC kit, Vector Laboratories Inc., Burlingame, CA, United States). The tissue microarray contains gastric tumor tissues and adjacent nontumor gastric tissues from 40 gastric cancer patients. The anti-S1PR1 antibody (55133-I-AP, Proteintech) was used at 1:500 dilution. After washing, biotinylated secondary antibody (MP-7500, Vector Inc.) was applied at 1:100 or 1:200 dilutions for 1 h. Color was developed with diaminobenzidine (SK-4100, Vector Inc.). Sections were counterstained with Mayer’s hematoxylin. IHC staining intensity was determined by using Celleste^TM^ Image Analysis Software. Staining intensity was scored as follows: 1 = weak staining, 2 = medium staining, and 3 = strong staining. The IHC staining score was determined by measuring both the percentage of cells that stained positive for S1PR1 and the staining intensity.

### Statistical Analysis

Statistical analysis was performed using the GraphPad Prism Statistics Program (Version 7, GraphPad Prism Software). All the data were obtained from experiments with adequate sample size and presented as means ± SE. Student’s *t*-tests were performed for comparisons between two groups. One-way ANOVA was carried out for comparisons among three or more groups and was followed by Tukey’s *post hoc* test. Values were considered to be significant at *p* < 0.05.

## Results

### Anticancer Drug-Resistant Gastric Cancer Cells Show Enhanced Autophagic Process and Antiapoptotic Effects

**FIGURE 1:**
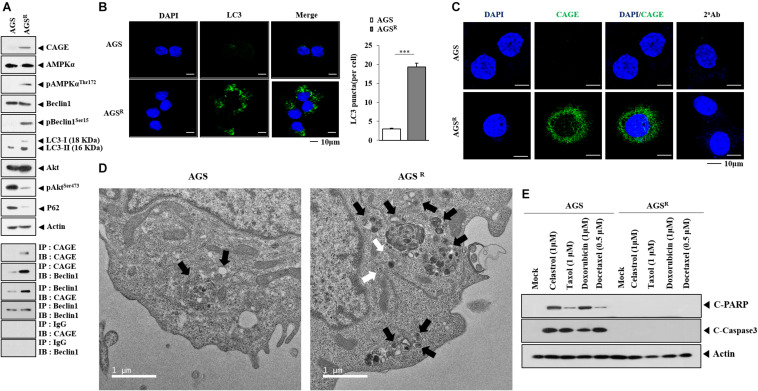
Anticancer drug resistance is correlated with enhanced autophagy and antiapoptotic effects. **(A)** Cell lysates were subjected to immunoblot and immunoprecipitation. Representative blots of three independent experiments are shown. Immunofluorescence staining of LC3 **(B)** and cancer-associated gene (CAGE) **(C)** was performed. ***, *p* < 0.001. **(D)** Representative electron micrograph of AGS cells and AGS^*R*^ cells is shown. The black arrows indicate autolysosomes. The white arrows indicate multivesicular bodies. **(E)** After 48 h of treatment, immunoblot was performed. C-PARP denotes cleaved PARP. Representative blots of three independent experiments are shown.

To better understand the mechanism of anticancer drug resistance, AGS^*R*^ (cells) were established. AGS^*R*^ cells showed higher resistance to various anticancer drugs compared with parental AGS cells (Table 1). AGS^*R*^ cells showed increased autophagic flux, including AMP-activated protein kinase (pAMPKa^*T172*^), pBeclin1^*Ser15*^, and LC3-II formation in comparison with AGS cells (Figure 1A). The increased LC3-II formation is a hallmark of autophagy (Klionsky et al., 2021). AGS^*R*^ cells showed an increased expression of CAGE compared with AGS cells (Figure 1A). AGS^*R*^ cells showed decreased levels of pAKt^*Ser473*^ and p62 compared with AGS cells (Figure 1A). CAGE was shown to confer anticancer drug resistance in melanoma cells (Kim et al., 2017a). In AGS^*R*^ cells, CAGE was shown to bind to Beclin1 (Figure 1A). AGS^*R*^ cells displayed an increased number of LC3 puncta (Figure 1B). CAGE showed localization in the nucleus and nuclear membrane (Figure 1C). AGS^*R*^ cells displayed an enhanced autophagic process when compared with AGS cells (Figure 1D). AGS cells, but not AGS^*R*^ cells, showed the cleavage of PARP and caspase-3 in response to various anticancer drugs (Figure 1E). Anticancer drug-resistant melanoma cells (Malme3M^*R*^) showed a higher expression of CAGE than the anticancer drug-sensitive Malme3M cells (Kim et al., 2017a). Malme3M^*R*^ cells, but not Malme3M cells, showed an increased expression of pBeclin1^*Ser15*^ and binding of CAGE to Beclin1 (Supplementary Figure 1A). CAGE induced the binding of CAGE to Beclin1 and inhibited the binding of Beclin1 to Bcl-2, an inhibitor of autophagy (Supplementary Figure 1B). Malme3M^*R*^ cells also displayed an enhanced autophagic process compared with Malme3M cells (Supplementary Figure 1C). Therefore, anticancer drug resistance is closely associated with antiapoptotic effects and autophagic flux.

**TABLE 1:** Anticancer drug resistance of AGS^*R*^ cells.

	IC_50_ (μ M)	
	
	AGS	AGS^*R*^
Celastrol	0.94 ± 0.09	1.96 ± 0.53
Taxol	0.29 ± 0.019	0.81 ± 0.03
Doxorubicin	0.05 ± 0.1	0.18 ± 0.09
Docetaxel	8.17 ± 0.76	10.06 ± 2.4

*The indicated cancer cell line was treated with the indicated drugs for 48 h. The cell viability was analyzed by an MTT assay. The IC_50_ value of each cell line was determined from the concentration–response curves. All values indicate the mean ± SD.*

### CAGE Regulates the Response to Anticancer Drugs and Autophagic Flux

**FIGURE 2:**
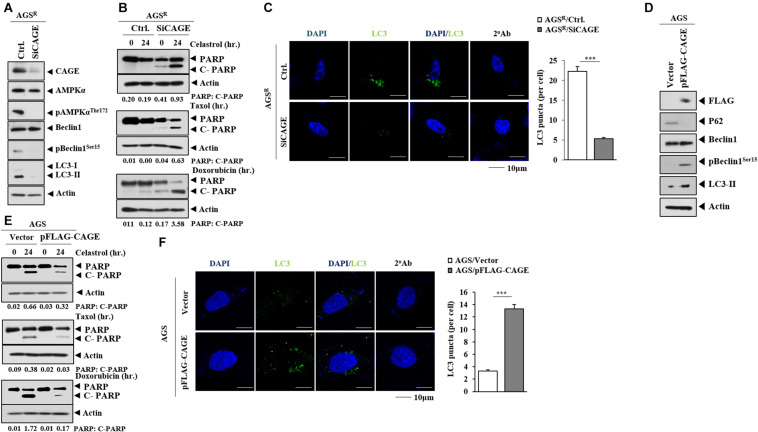
CAGE regulates anticancer drug resistance and autophagic flux. **(A)** After 48 h of transfection with the indicated siRNA (each at 10 nM), immunoblot was performed. Representative blots of three independent experiments are shown. Ctrl. denotes control siRNA. **(B)** After 24 h of transfection, cells were treated with the indicated anticancer drug for 24 h. Quantification of band intensity was represented by the ratio of cleaved/full-length PARP normalized to actin. **(C)** Same as **(A)** except that immunofluorescence staining was performed. ***, *p <* 0.001. **(D)** After 48 h of transfection with the indicated construct, immunoblot was performed. Representative blots of three independent experiments are shown. **(E)** After 24 h of transfection, the cells were treated with the indicated anticancer drug (each at 1 μM) for 24 h. **(F)** Same as **(D)** except for the immunofluorescence staining.

The decreased expression of CAGE decreased autophagic flux (Figure 2A) and the number of LC3 puncta (Figure 2C). The downregulation of CAGE enhanced the effects of anticancer drugs on the cleavage of PARP in AGS^*R*^ cells (Figure 2B). CAGE increased the expression of pBeclin1^*Ser15*^ and LC3-II formation but decreased the level of p62, a selective receptor of autophagy, in AGS cells (Figure 2D). CAGE inhibited the effects of anticancer drugs on the cleavage of PARP (Figure 2E) but increased the number of LC3 puncta in AGS cells (Figure 2F). CRISPR/Cas9 KO cell lines were established to further determine the role of CAGE in relation to autophagy and anticancer drug resistance. AGS^*R*△^ ^*CAGE#5*^ and AGS^*R*△^ ^*CAGE#7*^ cell lines showed decreased expression of CAGE, autophagic flux (Supplementary Figure 2A), and LC3 puncta compared with AGS^*R*^ cells (Supplementary Figure 2B). AGS^*R*△^ ^*CAGE#5*^ and AGS^*R*△^ ^*CAGE#7*^ cell lines showed a lower invasion potential than AGS^*R*^ cells (Supplementary Figure 2C). AGS^*R*△^ ^*CAGE#5*^ and AGS^*R*△^ ^*CAGE#7*^ cell lines showed decreased expression levels of SNAIL and vimentin compared with AGS^*R*^ cells (Supplementary Figure 2C). AGS^*R*△^ ^*CAGE#5*^ and AGS^*R*△^ ^*CAGE#7*^ cell lines showed enhanced sensitivity to anticancer drugs compared with AGS^*R*^ cells (Supplementary Figure 2D). AGS^*R*△^ ^*CAGE#5*^ and AGS^*R*△^ ^*CAGE#7*^ cell lines showed lower tumor spheroid-forming potential (Supplementary Figure 2E) and proliferation potential than AGS^*R*^ cells (Figure 2F). Overexpression of CAGE increased autophagic flux (Supplementary Figure 3A), invasion potential (Supplementary Figure 3B), proliferation potential (Supplementary Figure 3C), tumor spheroid-forming potential, and the expression of SRY-Box Transcription Factor 2 (SOX2), an indicator of cancer stemness (Supplementary Figure 3D); however, it decreased the apoptotic effects of anticancer drugs in AGS^*R*△^ ^*CAGE#5*^ and AGS^*R*△^ ^*CAGE#7*^ cell lines (Supplementary Figure 3E). Thus, CAGE regulates both anticancer drug resistance and autophagic flux.

### Autophagy Is Accompanied by Anticancer Drug Resistance and Binding of CAGE to Beclin1

**FIGURE 3:**
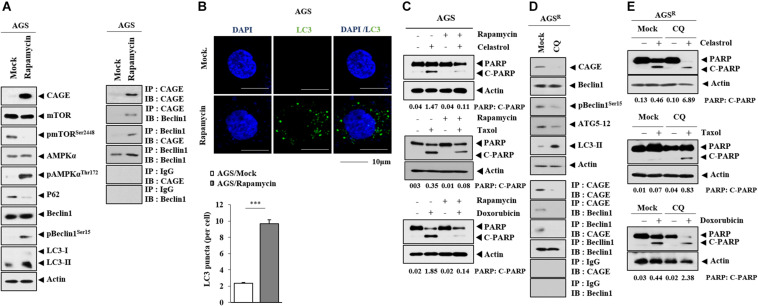
CAGE expression is regulated by autophagy regulators. **(A)** AGS cells were treated with or without rapamycin for 24 h. Representative blots of three independent experiments are shown. **(B)** AGS cells were treated with or without rapamycin for 24 h, followed by immunofluorescence staining. ***, *p* < 0.001. **(C)** AGS cells were treated with or without rapamycin (5 μM) for 24 h, followed by treatment with celastrol (1 μM), Taxol (1 μM), or doxorubicin (1 μM) for 24 h. Representative blots of three independent experiments are shown. **(D)** AGS^*R*^ cells were treated with or without chloroquine (CQ) (100 μM) for 24 h. Representative blots of three independent experiments are shown. **(E)** AGS^*R*^ cells were treated with or without CQ (100 μM) for 24 h, followed by treatment with the indicated anticancer drug for 24 h. Representative blots of three independent experiments are shown.

Rapamycin, an autophagy inducer, increased CAGE expression and autophagic flux but decreased p62 expression in AGS cells (Figure 3A). Rapamycin induced the binding of CAGE to Beclin1 (Figure 3A) and increased the number of LC3 puncta (Figure 3B). Rapamycin inhibited the effects of anticancer drugs on the cleavage of PARP (Figure 3C). Chloroquine (CQ), an inhibitor of autophagy, decreased the expression of CAGE, pBeclin1^*Ser15*^, and autophagy-related-5 (ATG5), but increased LC3-II formation in AGS^*R*^ cells (Figure 3D). CQ inhibited the interaction between CAGE and Beclin1 (Figure 3D). Additionally, CQ enhanced the effects of anticancer drugs on the cleavage of PARP (Figure 3E). Thus, increased autophagic flux is accompanied by the increased expression of CAGE.

### MiR-302b-5p Targets CAGE

**FIGURE 4:**
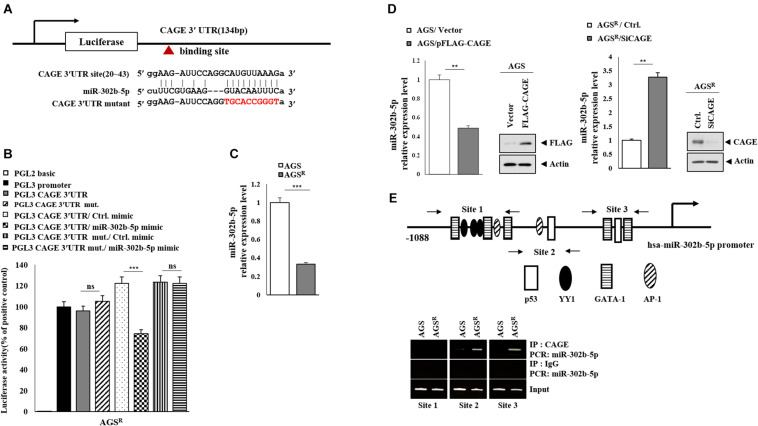
CAGE and miR-302b-5p form a negative feedback loop. **(A)** Shows the binding site for miR-302b-5p in the 3′ UTR of CAGE. **(B)** After 48 h of transfection, luciferase activity assays were performed as described. ***, *p* < 0.001. Average values of three independent experiments are shown. Ns denotes not significant. **(C)** qRT-PCR analysis was performed. ***, *p* < 0.001. Average values of three independent experiments are shown. **(D)** After 48 h of transfection, qRT-PCR and immunoblot were performed. **, *p* < 0.01. Average values of three independent experiments are shown. **(E)** ChIP assays were performed as described.

MiR-302b-5p was predicted to bind to the 3′ UTR of CAGE (Figure 4A). The miR-302b-5p mimic decreased the luciferase activity of wild-type CAGE 3′ UTR, but not the luciferase activity of the mutant CAGE 3′ UTR (Figure 4B). AGS^*R*^ cells showed a lower expression of miR-302b-5p compared with AGS cells (Figure 4C). CAGE decreased miR-302b-5p expression level in AGS cells (Figure 4D), while the decreased expression of CAGE increased miR-302b-5p expression in AGS^*R*^ cells (Figure 4D). MiR-302b-5p promoter displays the potential binding sites for p53, AP1, YY1, and GATA-1 (Figure 4E). CAGE was shown to bind to the promoter sequences of miR-302b-5p in chromatin immunoprecipitation (ChIP) assays (Figure 4E). Thus, CAGE and miR-302b-5p cross-regulate each other. It is likely that miR-302b-5p may regulate autophagic flux.

### MiR-302b-5p Regulates Autophagic Flux

**FIGURE 5:**
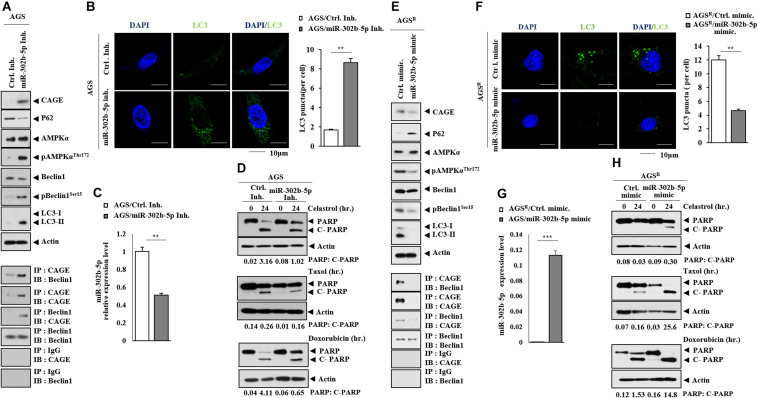
MiR-302b-5p regulates autophagic flux and anticancer drug resistance. **(A)** After 48 h of transfection with the indicated inhibitor (each at 10 nM), immunoblot and immunoprecipitation were performed. Representative blots of three independent experiments are shown. **(B)** Same as **(A)** except that immunofluorescence staining was performed. **, *p* < 0.01. **(C)** qRT-PCR was performed. **, *p* < 0.01. Average values of three independent experiments are shown. **(D)** AGS cells were transfected with the indicated inhibitor (each at 10 nM). The following day, cells were then treated with or without celastrol (1 μM), Taxol (1 μM), or doxorubicin (1 μM) for 24 h. Representative blots of three independent experiments are shown. **(E)** After 48 h of transfection with the indicated mimic (each at 10 nM), immunoblot and immunoprecipitation were performed. Representative blots of three independent experiments are shown. **(F)** Same as **(E)** except that the number of LC3 puncta was determined. **, *p* < 0.01. **(G)** qRT-PCR analysis was performed. ***, *p* < 0.001. Average values of three independent experiments are shown. **(H)** AGS^*R*^ cells were transfected with the indicated mimic (each at 10 nM). The next day, cells were then treated with or without celastrol (1 μM), Taxol (1 μM), or doxorubicin (1 μM) for 24 h. Representative blots of three independent experiments are shown.

The miR-302b-5p inhibitor increased the expression of CAGE, autophagic flux, the binding of CAGE to Beclin1 (Figure 5A), and the number of LC3 puncta (Figure 5B). The miR-302b-5p inhibitor decreased miR-302b-5p expression in AGS cells (Figure 5C). The miR-302b-5p inhibitor inhibited the effects of anticancer drugs on the cleavage of PARP (Figure 5D). The miR-302b-5p mimic decreased CAGE expression and autophagic flux but increased p62 level (Figure 5E). In AGS^*R*^ cells, the miR-302b-5p mimic inhibited CAGE binding to Beclin1 (Figure 5E). MiR-302b-5p mimic decreased the number of LC3 puncta in AGS^*R*^ cells (Figure 5F). Transfection of miR-302b-5p mimic increased miR-302b-5p expression in AGSR cells (Figure 5G). MiR-302b-5p mimic enhanced the effects of anticancer drugs on the cleavage of PARP (Figure 5H). Thus, miR-302b-5p regulates autophagic flux and anticancer drug resistance through a negative feedback loop with CAGE.

### Soluble Factors Regulate Autophagic Flux

**FIGURE 6:**
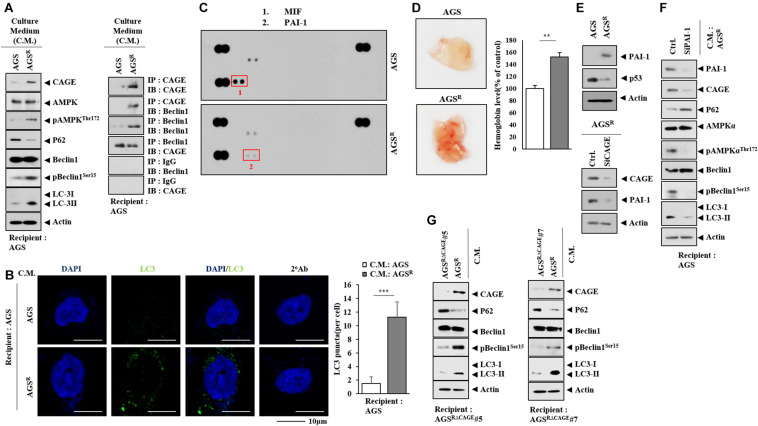
Soluble factors regulate autophagic flux. **(A)** Culture medium of AGS^*R*^ cells were added to AGS cells for 24 h. Representative blots of three independent experiments are shown. **(B)** Same as **(A)** except that the number of LC3 puncta was determined. ***, *p* < 0.001. **(C)** Cytokine array analysis was performed. **(D)** Matrigel plug assays using culture medium were performed. **(E)** Immunoblot was performed. Representative blots of three independent experiments are shown. **(F)** After 48 h of transfection with the indicated siRNA (each at 10 nM), culture medium was added to AGS cells for 24 h. Representative blots of three independent experiments are shown. **(G)** Culture medium of the indicated cancer cells was added to the CAGE CRISPR–Cas9 cell line for 24 h. Representative blots of three independent experiments are shown.

Next, we examined whether anticancer drug resistance could be transferred. The culture medium from AGS^*R*^ cells increased the expression of CAGE and autophagic flux and induced CAGE binding to Beclin1 in AGS cells (Figure 6A). The culture medium from AGS^*R*^ cells, but not from AGS cells, increased the number of LC3 puncta in AGS cells (Figure 6B). The culture medium of AGS^*R*^ cells showed a higher expression of PAI-1 than that of AGS cells (Figure 6C). PAI-1 has been shown to promote angiogenesis (Park et al., 2014). The culture medium from AGS^*R*^ also showed an enhanced angiogenic potential compared with the AGS cell culture medium based on Matrigel plug assays (Figure 6D). AGS^*R*^ cells displayed a higher expression of PAI-1 than AGS cells (Figure 6E). The downregulation of CAGE led to decreased expression of PAI-1 in AGS^*R*^ cells (Figure 6E). PAI-1 was necessary for increased CAGE expression and autophagic flux in AGS cells by culture medium of AGS^*R*^ cells (Figure 6F). The culture medium from AGS^*R*^ cells increased autophagic flux in CAGE CRISPR–Cas9 cell lines (Figure 6G). The CAGE-derived AQTGTGKT peptide inhibited CAGE binding to Beclin1 and enhanced the sensitivity to anticancer drugs in nonsmall cell lung cancer cells and melanoma cells, respectively (Kim et al., 2017a; Yeon et al., 2018). The AQTGTGKT negatively regulated autophagic flux and inhibited CAGE binding to Beclin1 in AGS^*R*^ cells (Supplementary Figure 4A) and was shown to bind to CAGE in AGS^*R*^ cells (Supplementary Figure 4B) and decreased the number of LC3 puncta (Supplementary Figure 4C). The culture medium from AGS^*R*^ cells that were treated with the AQTGTGKT peptide did not increase the autophagic flux in AGS cells (Supplementary Figure 4D). GW4869, an inhibitor of exosome formation, inhibited the effect of the culture medium of AGS^*R*^ cells on autophagic flux in AGS cells (Supplementary Figure 5A). Also, GW4869 inhibited the effect of the culture medium of AGS^*R*^ cells on the number of LC3 puncta (Supplementary Figure 5B). These results suggest that soluble factors may regulate autophagic flux and anticancer drug resistance.

### Exosomes Regulate Autophagic Flux and the Response to Anticancer Drugs

**FIGURE 7:**
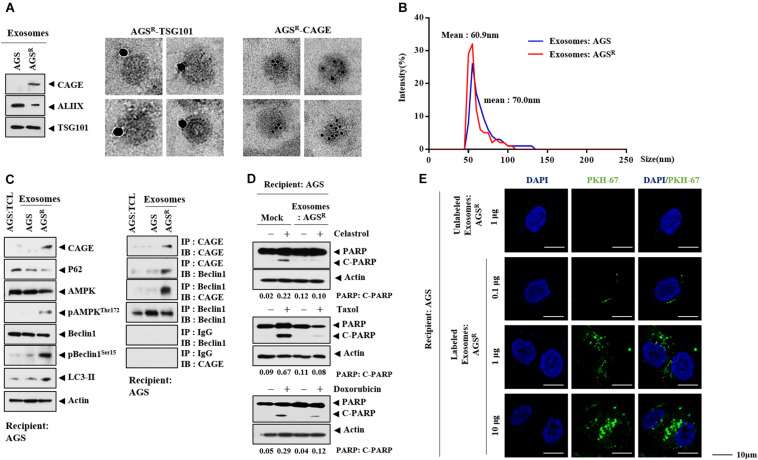
Exosomes of AGS^*R*^ cells enhance autophagic flux and decrease the PARP cleavage. **(A)** Immunoblot (left) and ImmunoEM (left) show the presence of CAGE in the exosomes. Immunogold staining images using anti-TSG101 and anti-CAGE antibody are shown. Twenty-five and 10 nm gold particles are used to indicate the localization of TSG 101 and CAGE, respectively. CAGE is present in the lumen of the exosomes. **(B)** Size distribution of exosomes is shown. **(C)** Exosomes (10 μg/ml) of the indicated cancer cells were added to AGS cells for 24 h. TCL denotes total cell lysates. Representative blots of three independent experiments are shown. **(D)** AGS cells were treated with or without exosomes (10 μg/ml) of AGS^*R*^ cells for 24 h, followed by treatment with the indicated anticancer drug for 24 h. Representative blots of three independent experiments are shown. **(E)** Exosomes (10 μg/ml) of AGS^*R*^ cells were labeled with or without PKH67. Exosomes were then added to AGS cells for 24 h.

CAGE was shown to be present in the sera of various cancer patients (Cho et al., 2003). This implies the presence of CAGE in exosomes. Immunoblot and ImmunoEM revealed the presence of CAGE in the exosomes of AGS^*R*^ cells (Figure 7A). Figure 7B shows the size distribution of exosomes. Exosomes from AGS^*R*^ cells increased CAGE expression and autophagic flux and induced the CAGE binding to Beclin1 in AGS cells (Figure 7C). Exosomes from AGS^*R*^ cells inhibited the effects of anticancer drugs on the cleavage of PARP (Figure 7D). PKH labeling showed the internalization of the exosomes from AGS^*R*^ cells into AGS cells (Figure 7E). GW4869 inhibited the effects of the culture medium from Malme3M^*R*^ cells on the expression of CAGE expression and autophagic flux in Malme3M cells (Supplementary Figure 6A). GW4869 inhibited the effects of the culture medium of Malme3M^*R*^ cells on the cleavage of PARP and FAK in Malme3M cells (Supplementary Figure 6B). ImmunoEM (Supplementary Figure 6C) and immunoblot (Supplementary Figure 6D) analyses revealed the presence of CAGE within the exosomes. Human recombinant CAGE protein increased autophagic flux and induced CAGE binding to Beclin1 in AGS cells (Supplementary Figure 7A) and enhanced the invasion potential of AGS cells (Supplementary Figure 7B), in addition to increasing the number of LC3 puncta (Supplementary Figure 7C). Human recombinant CAGE protein prevented CQ from decreasing autophagic flux (Supplementary Figure 7D). Human recombinant CAGE protein inhibited the effects of anticancer drugs on the PARP cleavage in AGS cells (Supplementary Figure 7E). Thus, CAGE may act as a soluble mediator of anticancer drug resistance.

### MiR-181-5p Negatively Regulates the Expression of CAGE and Autophagic Flux

**FIGURE 8:**
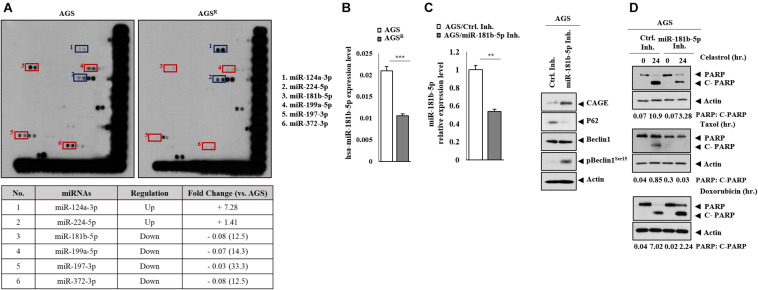
MiR-181b-5p negatively regulates the expression of CAGE, autophagic flux. **(A)** MicroRNA array analysis was performed. The red rectangles show miRNAs expressed highly in AGS cells. The blue rectangles show miRNAs expressed highly in AGS^*R*^ cells. **(B)** qRT-PCR analysis was performed. ***, *p* < 0.001. Average values of three independent experiments are shown. **(C)** After 48 h of transfection with the indicated inhibitor (each at 10 nM), qRT-PCR and immunoblot were performed. **, *p* < 0.01. Average values of three independent experiments are shown. **(D)** AGS cells were transfected with the indicated inhibitor (each at 10 nM). The following day, the cells were then treated with or without celastrol (1 μM) or Taxol (1 μM) for 24 h, followed by immunoblot. Representative blots of three independent experiments are shown.

MiRNAs that were differentially expressed between AGS and AGS^*R*^ cells were identified by miRNA array analysis Figure 8A). MiR-181-b-5p was one of those miRNAs that could act as a negative regulator of anticancer drug resistance (Figure 8A). qRT-PCR showed a higher expression of miR-181b-5p in AGS cells in comparison with AGS^*R*^ cells (Figure 8B). The miR-181b-5p inhibitor increased CAGE expression and pBeclin1^*Ser15*^ but decreased p62 level (Figure 8C). The miR-181b-5p inhibitor negatively regulated the effects of anticancer drugs on the cleavage of PARP in response to anticancer drugs in AGS cells (Figure 8D).

### S1PR1 Serves as a Target of MiR-181b-5p

**FIGURE 9:**
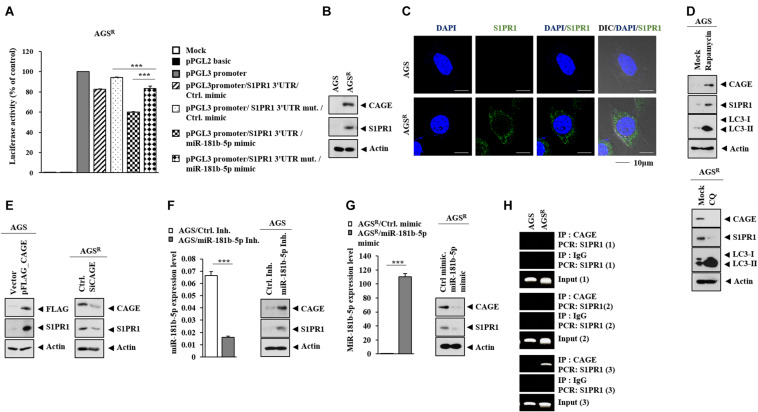
SIPR1 serves as a target of miR-181b-5p. **(A)** After 48 h of transfection with the indicated construct (each at 1 μg) and/or mimic (each at 10 nM), luciferase activity assays were performed. ***, *p* < 0.001. Average values of three independent experiments are shown. **(B)** Immunoblot was performed. Representative blots of three independent experiments are shown. **(C)** Cytoplasmic localization of S1PR1 is shown. **(D)** The indicated cancer cells were treated with or without rapamycin (5 μM) or CQ (100 μM) for 24 h. Representative blots of three independent experiments are shown. **(E)** After 48 h of transfection, immunoblot was performed. Representative blots of three independent experiments are shown. **(F)** After 48 h of transfection, qRT-PCR and immunoblot were performed. ***, *p* < 0.001. Average values of three independent experiments are shown. **(G)** After 48 h of transfection, qRT-PCR and immunoblot were performed. ***, *p* < 0.001. Average values of three independent experiments are shown. **(H)** ChIP assays were performed as described.

S1PR1 was predicted as a potential target of miR-181b-5p in TargetScan analysis. The miR-181-b-5p mimic decreased the luciferase activity of the wild-type 3′ UTR of S1PR1, but not the luciferase activity of the mutant 3′ UTR of S1PR1 (Figure 9A). AGS^*R*^ cells showed a higher expression of S1PR1 compared with AGS cells (Figure 9B). S1PR1 was localized in the cytoplasm of AGS^*R*^ cells (Figure 9C). Rapamycin increased S1PR1 expression in AGS cells, while CQ decreased S1PR1 expression in AGS^*R*^ cells (Figure 9D). CAGE (Figure 9E), miR-181b-5p inhibitor (Figure 9F), and miR-181b-5p mimic (Figure 9G) regulated S1PR1 expression. CAGE was shown to bind to the promoter sequences of S1PR1 in ChIP assays (Figure 9H). Thus, the CAGE–miR-181b-5p–S1PR1 axis and autophagy cross-regulate each other.

### S1PR1 Regulates Autophagic Flux and the Response to Anticancer Drugs

**FIGURE 10:**
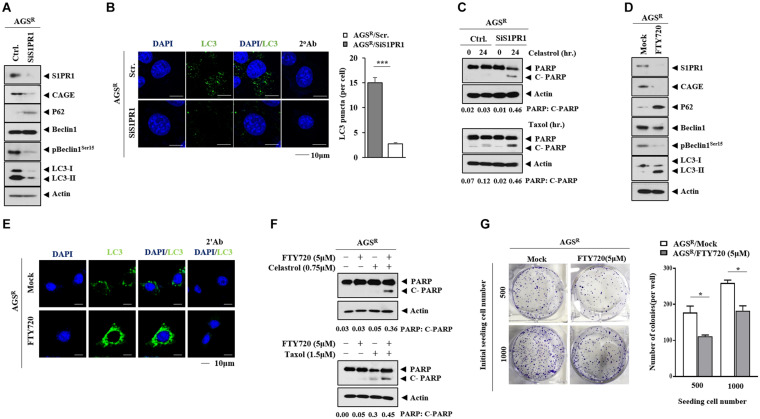
S1PR1 regulates autophagic flux and anticancer drug resistance. **(A)** After 48 h of transfection, immunoblot was performed. Representative blots of three independent experiments are shown. **(B)** The number of LC3 puncta was determined. ***, *p* < 0.001. **(C)** After 24 h of transfection, cells were then treated with or without the indicated anticancer drug for 24 h. Representative blots of three independent experiments are shown. **(D)** The indicated cancer cells were treated with or without FTY720 (5 μM) for 24 h. Representative blots of three independent experiments are shown. **(E)** Immunofluorescence staining was performed. **(F)** AGS^*R*^ cells were treated with or without FTY720 (5 μM) for 24 h. Cells were then treated with the anticancer drug for 24 h. Representative blots of three independent experiments are shown. **(G)** Colony-forming potential was determined. *, *p* < 0.05. Average values of three independent experiments are shown.

The expression of S1PR1 in nontumor gastric tissues and gastric cancer tissues was examined. The GEPIA database showed that gastric cancer tissues had a higher expression of *SIPR1* mRNA than nontumor gastric tissues (Supplementary Figure 8A). Gastric cancer tissues showed higher expression of SIPR1 compared with other nontumor gastric tissues (Supplementary Figures 8B,C). A high expression level of S1PR1 was correlated with low survival rates in gastric cancer patients (Supplementary Figure 8D). The downregulation of S1PR1 decreased CAGE expression and autophagic flux but increased p62 level in AGS^*R*^ cells (Figure 10A). The downregulation of S1PR1 resulted in a decreased number of LC3 puncta (Figure 10B), while the effects of anticancer drugs on the cleavage of PARP were enhanced (Figure 10C). The inhibition of S1PR1 by FTY720 decreased the expressions of CAGE and pBeclin1^*Ser15*^, but increased p62 level and LC3-II formation in AGS^*R*^ cells (Figure 10D). FTY720 increased the number of LC3 puncta in AGS^*R*^ cells (Figure 10E). Just like CQ, FTY720 may inhibit the fusion of autophagosomes and lysosomes. FTY enhanced the effects of anticancer drugs on the cleavage of PARP (Figure 10F). FTY720 decreased the colony-forming potential of AGS^*R*^ cells (Figure 10G). These results indicate that CAGE and S1PR1 form a positive feedback loop to regulate anticancer drug resistance and autophagic flux.

## Discussion

Anticancer drug-resistant gastric cancer cells cells displayed an increased expression of CAGE and autophagic flux compared with AGS cells (Figure 1A). Autophagy may protect tumor cells from chemotherapeutic drugs by inhibiting apoptotic cell death (Liu et al., 2018). The inhibition of autophagy by CQ enhances apoptosis (Pan et al., 2019). CAGE regulated autophagic flux (Figures 2A,D). CAGE has been shown to regulate autophagic flux in nonsmall cell lung cancer cells (Yeon et al., 2018). The downregulation of CAGE enhanced the effects of anticancer drugs on the cleavage of PARP in AGS^*R*^ cells (Figure 2B). This suggests that CAGE may affect the response to anticancer drugs by regulating autophagic flux. MAPK activation results in the increased autophagic flux in EGFR tyrosine kinase inhibitor-resistant nonsmall cell lung cancer cells (Lotsberg et al., 2020). MAPK activation may lead to increased CAGE expression in AGS^*R*^ cells. In future studies, it may be necessary to identify downstream targets of CAGE.

CAGE enhances the self-renewal activity of breast cancer cells (Kim et al., 2017c). Also, CAGE enhances the self-renewal and tumorigenic potential of Malme3M^*R*^ cells through interaction with SOX2 (Kim et al., 2017b). AGS^*R*^ cells displayed more enhanced tumor spheroid-forming potential compared with AGS cells (Supplementary Figure 2E). The binding of CAGE to SOX2 may lead to enhanced tumor spheroid-forming potential in AGS^*R*^ cells.

Luciferase activity assays demonstrate that miR-302b-5p acts as a negative regulator of CAGE (Figure 4B). MiR-302b-5p regulated both the autophagic flux (Figures 5A,E) and the response to anticancer drugs (Figures 5D,H). MiR-302 members enhance the chemosensitivity of breast cancer cells by decreasing breast cancer resistance protein (BCRP) expression and may cooperatively downregulate BCRP expression to increase the chemosensitivity of breast cancer cells (Wang et al., 2016). MiR-302b suppresses gastric cancer cell tumorigenesis and metastasis by regulating the EphA2/Wnt/β-catenin/EMT pathway (Huang et al., 2017). Overexpression of CAGE activates Wnt1 signaling in colon cells (Yeon et al., 2019). MiR-302b suppresses cell proliferation by inhibiting the effects on the increased expression levels of TGFβRII, phosphorylated ERK1/2, and MMP9 by TGF-β1 (Li et al., 2016). MiR-302b-3p targets IGF-1R and decreases the expression of cell cycle regulators such as cyclin D1 and CDK6 (Guo et al., 2017). MiR-302-367 cell-to-cell transfer decreases the expression levels of CXCR4/SDF1, SHH, cyclin D, cyclin A, and E2F1 to inhibit glioblastoma growth (Fareh et al., 2017). MiR-302 directly represses cyclin D1 and suppresses the proliferation of glioblastoma cells (Debruyne et al., 2018). Other downstream targets of miR-302b may regulate autophagic flux and anticancer drug resistance.

AGS^*R*^ cells revealed an increased expression of PAI-1 compared with AGS cells (Figure 6D). PAI-1 mediates pyruvate-induced angiogenesis (Jung et al., 2011) and serves as a target of HDAC3, thereby acting as an angiogenic factor (Park et al., 2014). AGS^*R*^ cells displayed a lower expression of HDAC3 than AGS cells (personal observations). The downregulation of PAI-1 decreased the expression levels of LC3 and Beclin1 (Wang et al., 2014). The culture medium of AGS^*R*^ cells increased autophagic flux in AGS cells in a PAI-1-dependent manner (Figure 6F). PAI-1 promotes the proliferation of head and neck cancer tumor-initiating cells (TICs) by increasing SOX2 expression (Lee et al., 2016). The effects of PAI-1 on the cancer stem cell-like properties of gastric cancer cells call for further investigation.

Soluble factors may regulate autophagic flux and anticancer drug resistance (Figure 6A). The AQTGTGKT peptide inactivates CAGE and prevents the culture medium of AGS^*R*^ cells from enhancing the autophagic flux in AGS cells (Figure S4D). This suggests that soluble factors may regulate autophagic flux in a CAGE-dependent manner. Exosomes promote autophagic flux in an AMPK-dependent manner (Zeng et al., 2020) and also confer resistance to anticancer drugs (Chinnappan et al., 2020). Gastric cancer-derived exosomes remodel the tumor microenvironment and affect anticancer drug resistance (Huang et al., 2019). Next, we examined the presence of CAGE in the exosomes. CAGE was found to be present in the exosomes of AGS^*R*^ cells (Figure 7A) and Malme3M^*R*^ cells (Supplementary Figure 6C). Exosomes from AGS^*R*^ cells increased autophagic flux (Figure 7C) and exerted antiapoptotic effects (Figure 7C). Thus, exosomal CAGE protein may confer resistance to anticancer drugs and further enhance autophagic flux. Exosomes from Taxol-resistant nasopharyngeal cancer cells show the presence of CAGE (Yuan and Zhou, 2021). In future studies, identification of miRNAs and soluble factors that could present differential expression in exosomes of AGS and AGS^*R*^ cells will be necessary.

A high miR-181b level results in an improved prognosis of human lung adenocarcinomas (Tan et al., 2018). The miR-181 family regulates VCAM-1 expression, and a low level of miR-181b is observed in high-grade glioma patients (Liu et al., 2017). MiR-181b-5p inhibits cellular invasion by regulating the expression of S1PR1 (Miao et al., 2020). AGS^*R*^ cells showed a lower expression of miR-181b-5p compared with AGS cells (Figures 8A,B). The miR-181b-5p inhibitor increased CAGE expression and autophagic flux in AGS cells (Figure 8C). CAGE did not affect the expression of miR-181b-5p (personal observations), implying that CAGE functions downstream of miR-181b-5p. It is possible that miR-181b-5p can predict the survival rate of gastric cancer patients.

MiR-181b-5p directly regulated the expression of S1PR1 (Figure 9A). FTY720, an inhibitor of S1PR1, overcomes resistance to ErbB inhibitors, such as afatinib (Booth et al., 2019). A high expression of S1PR1 predicts poor prognosis in breast cancer patients (Pyne et al., 2016) and gastric cancer patients (Supplementary Figure 7D). FTY720 decreased the expression of CAGE and autophagic flux (Figure 10D). It is necessary to examine the effect of sphingosine 1-phosphate on autophagic flux and identify additional targets of S1PR1. Sphingosine 1-phosphate may regulate autophagic flux and the identification of additional targets of S1PR1 is necessary.

In this study, we investigated the mechanism of CAGE-promoted anticancer drug resistance. Most importantly, we identified a novel role of the CAGE–miR-181b-5p–S1PR1 axis in the anticancer drug resistance and autophagic process. We show that CAGE can be employed as a target for the development of anticancer drugs.

## Data Availability Statement

The original contributions presented in the study are included in the article/Supplementary Material, further inquiries can be directed to the corresponding author/s.

## Ethics Statement

The animal study was reviewed and approved by the Institutional Animal Care and Use Committee (IACUC) of Kangwon National University. Written informed consent was obtained from the owners for the participation of their animals in this study.

## Author Contributions

DJ and YK provided the idea. DJ wrote the manuscript. MY performed most of the experiments in this study. DP, EK, and DK performed the expression and purification of CAGE protein. MJ and HJ performed the electron microscopy experiments. All authors contributed to the article and approved the submitted version.

## Conflict of Interest

The authors declare that the research was conducted in the absence of any commercial or financial relationships that could be construed as a potential conflict of interest.

## Footnotes

1 http://www.targetscan.org

2 http://diana.imis.athena-innovation.gr

3 http://mirdb.org
