# Supplementary material for: The CAGE–MiR-181b-5p–S1PR1 Axis Regulates Anticancer Drug Resistance and Autophagy in Gastric Cancer Cells
Source: Front Cell Dev Biol. 2021 May 25;9:666387. doi: 10.3389/fcell.2021.666387 (PMC8185229; doi:10.3389/fcell.2021.666387)

Supplementary Data

Each graph is densitometry analysis of western blots in this study. The levels of proteins were normalized to the level of Actin.

Figure 1

A

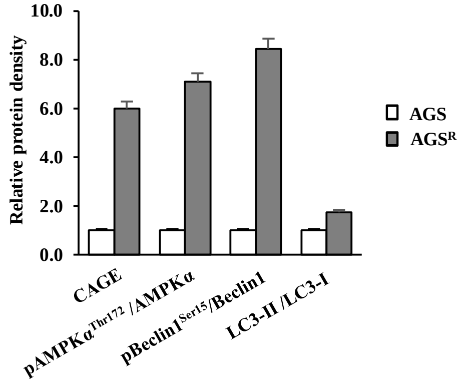

E

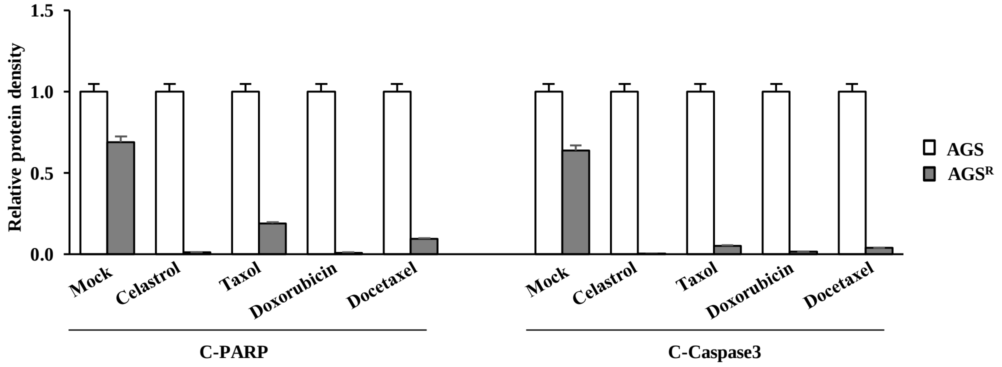

Figure 2

A

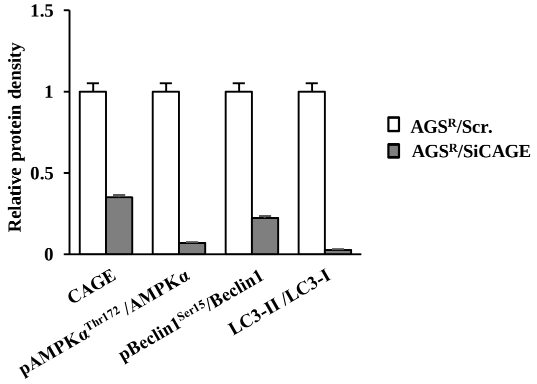

B

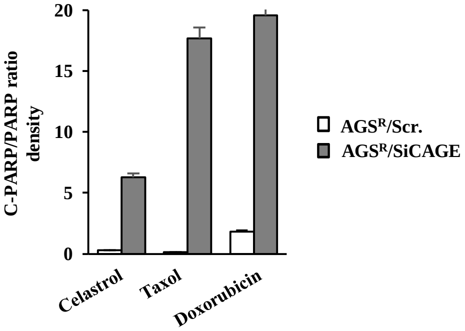

D

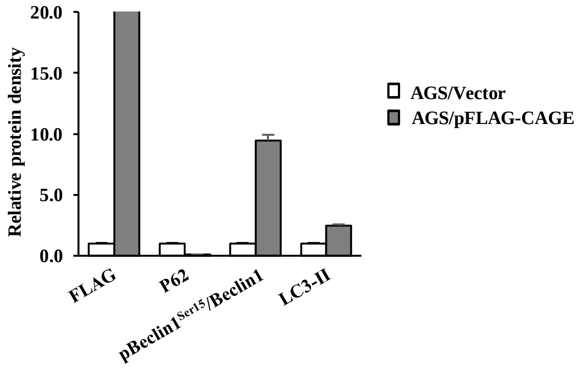

E

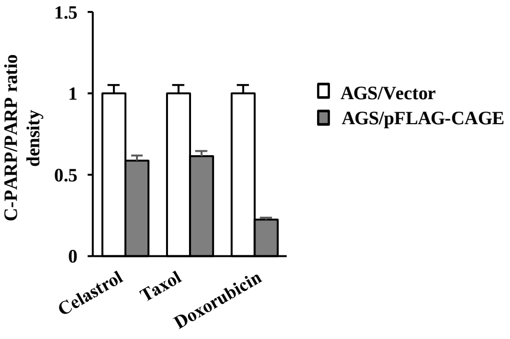

Figure 3

A

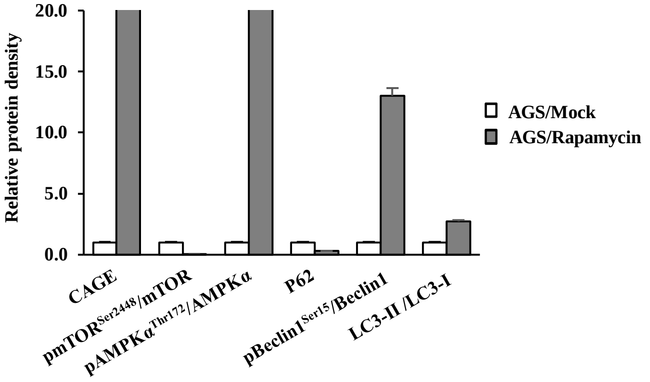

C

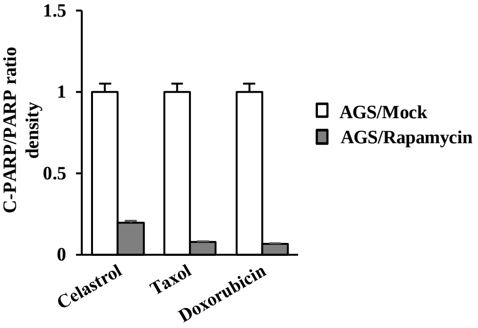

D

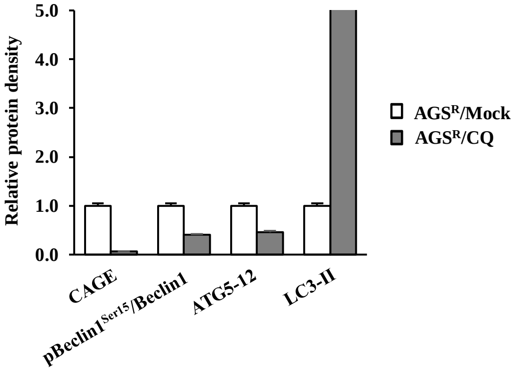

E

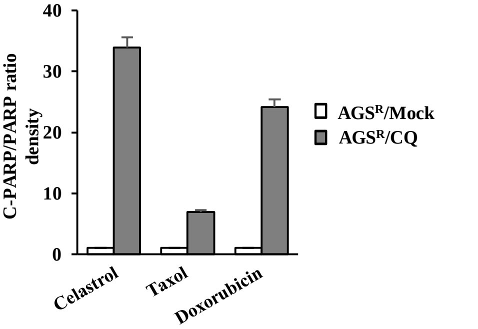

Figure 4

D

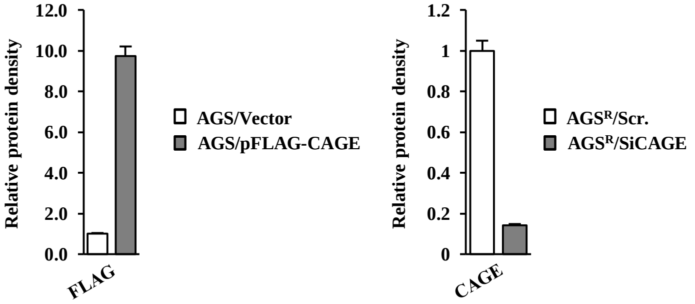

Figure 5

A

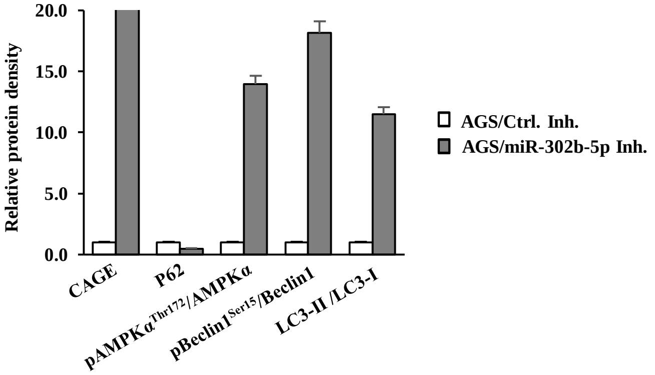

D

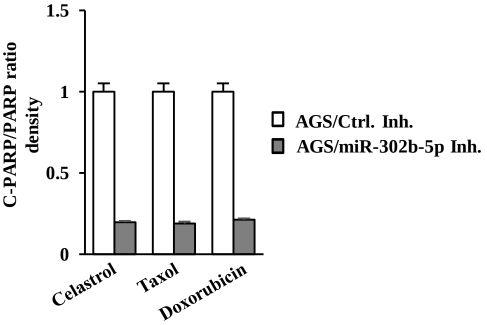

E

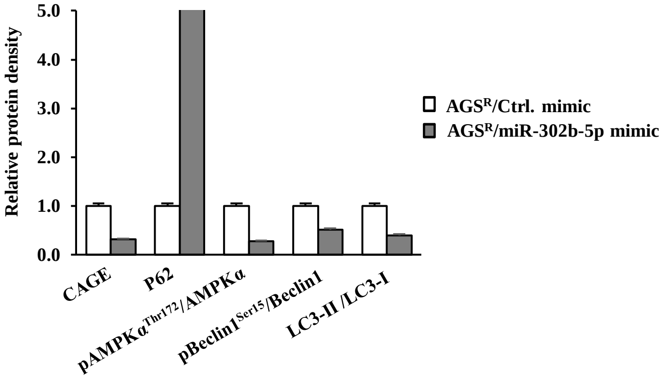

H

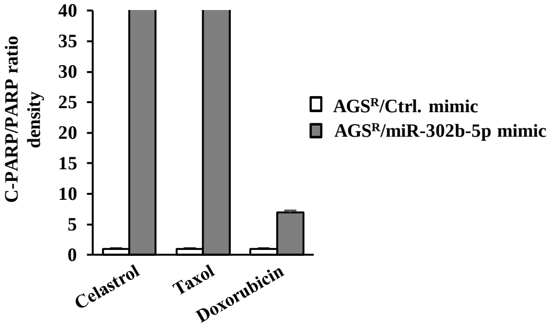

Figure 6

A

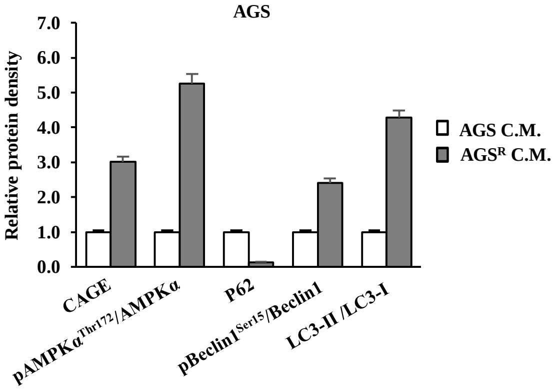

E

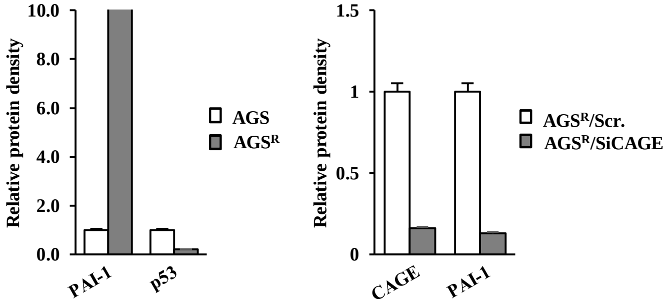

F

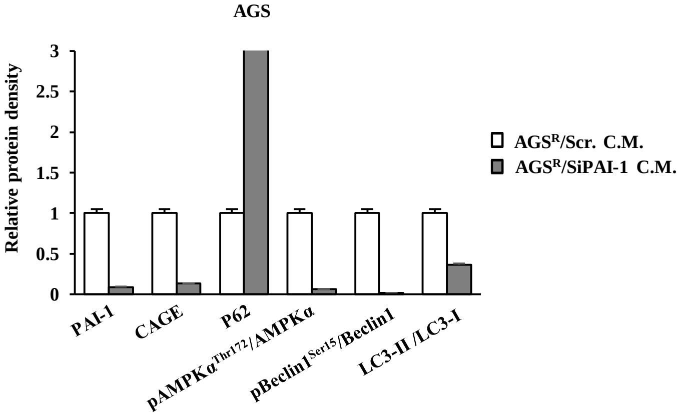

G

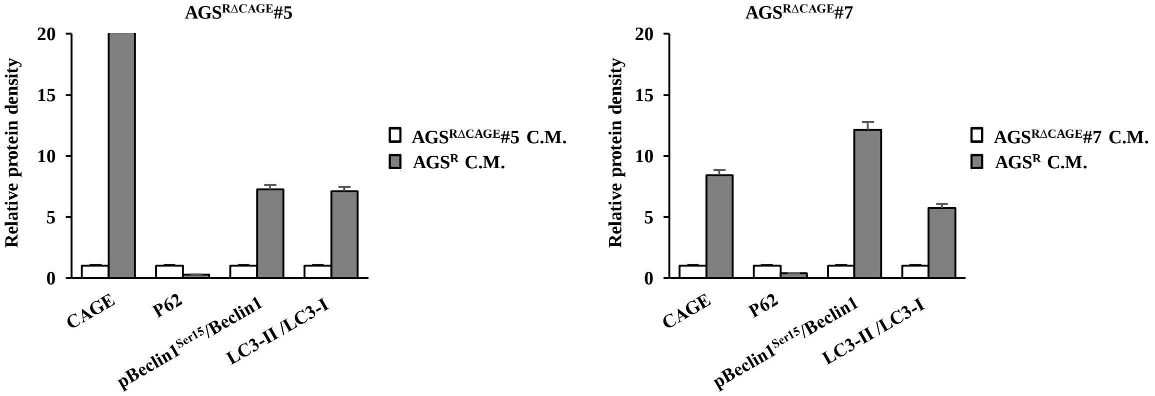

Figure 7

A

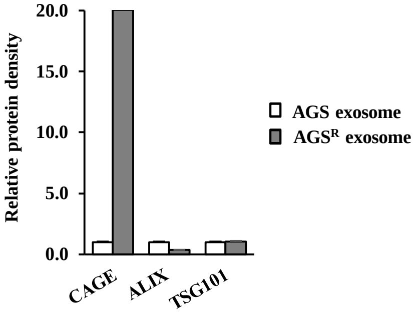

C

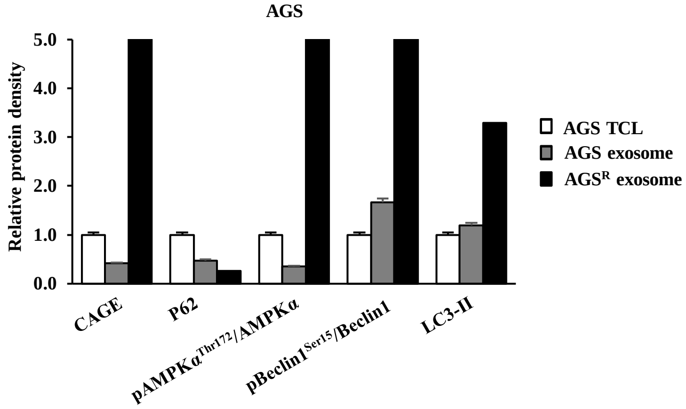

D

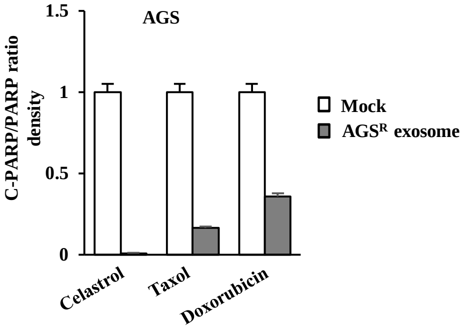

Figure 8

C

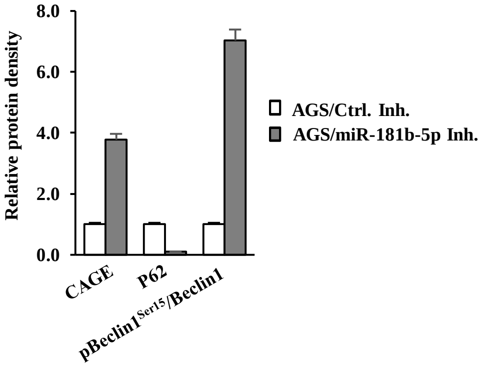

D

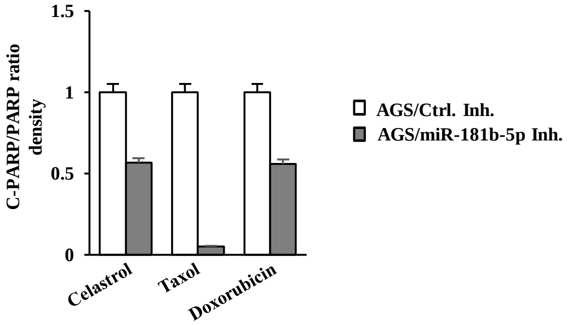

Figure 9

B

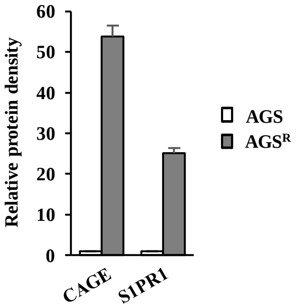

D

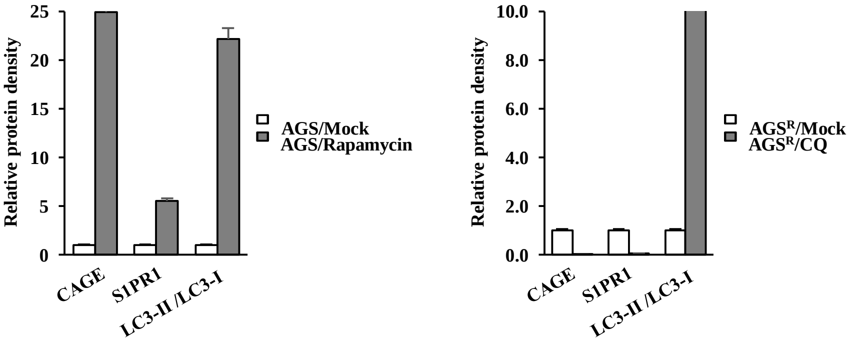

E

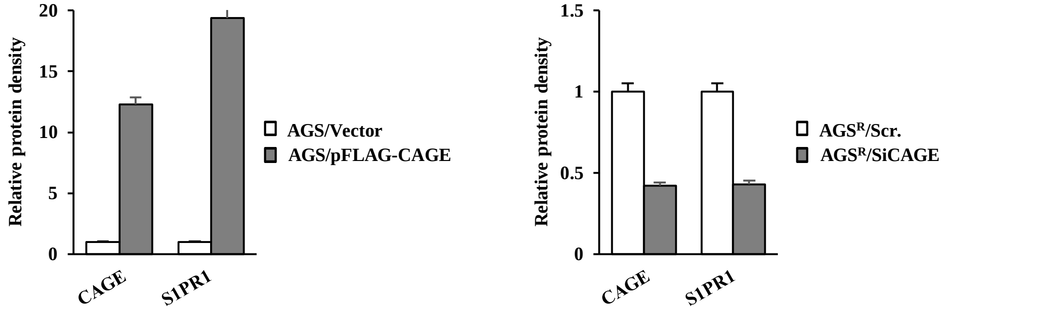

F

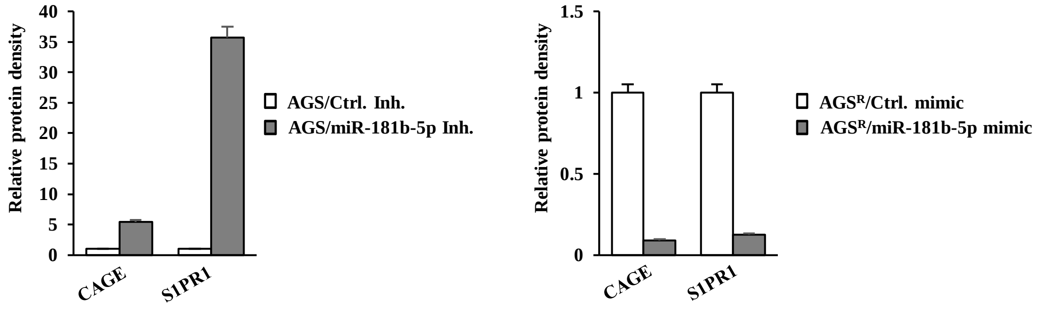

Figure 10

A

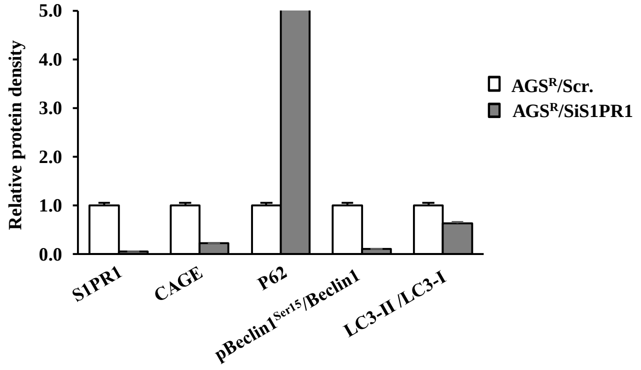

C

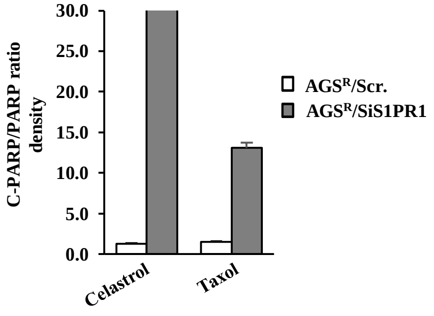

D

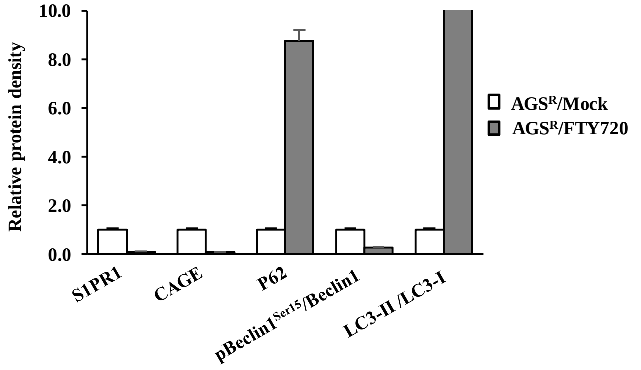

F

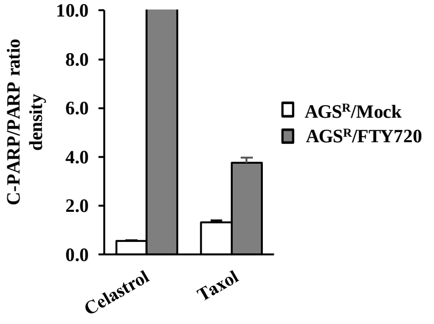

Figure S1

A

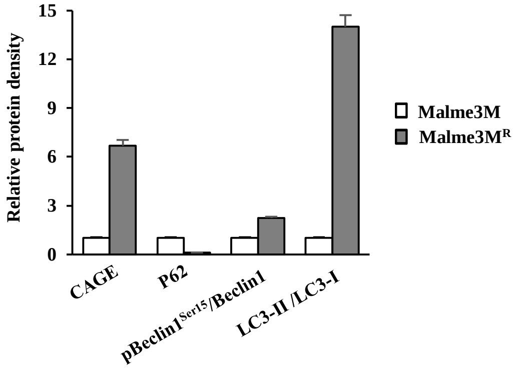

Figure S2

A

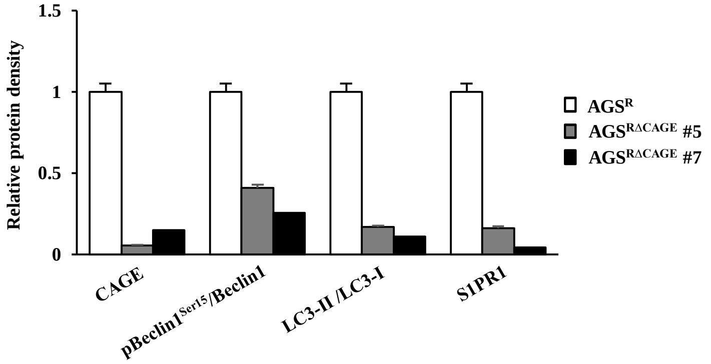

C

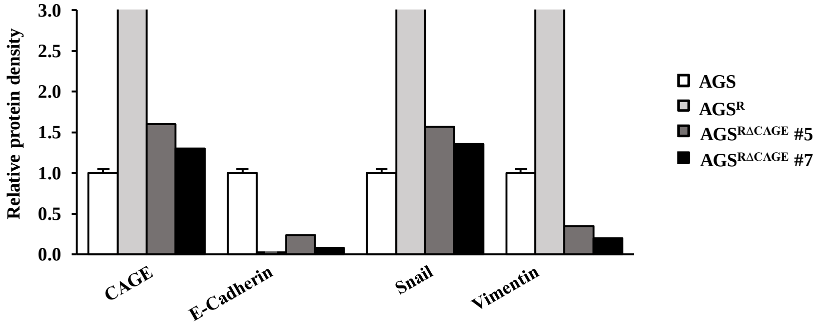

Figure S3

A

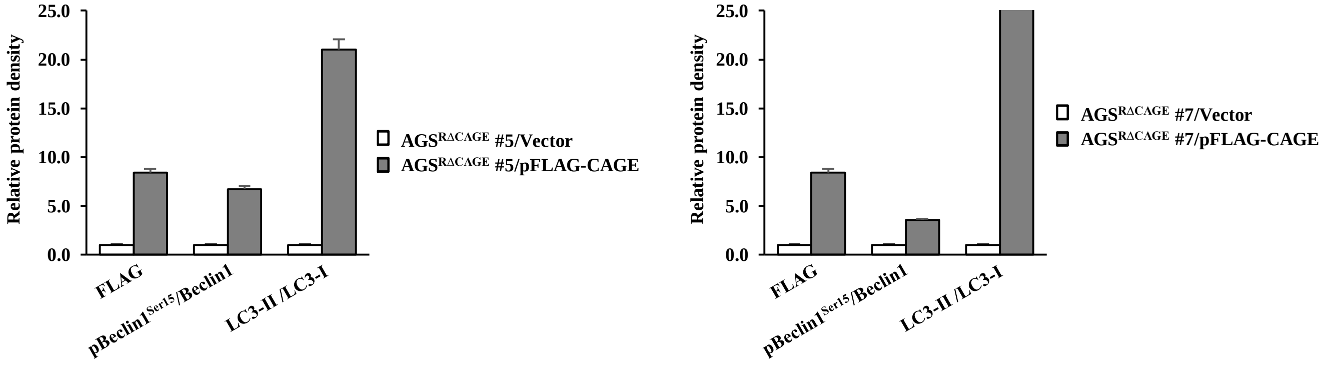

B

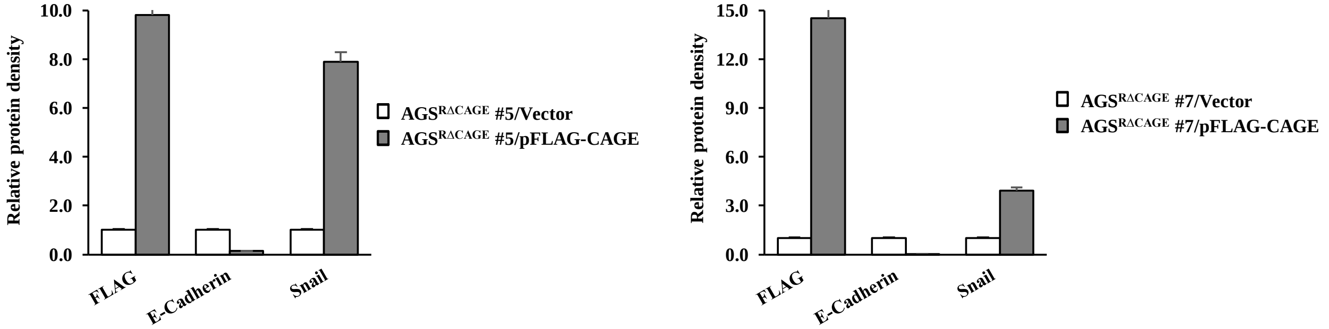

E

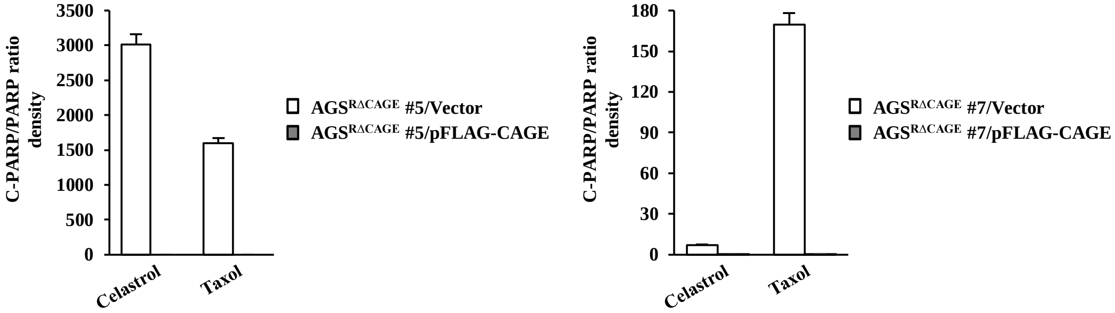

Figure S4

A

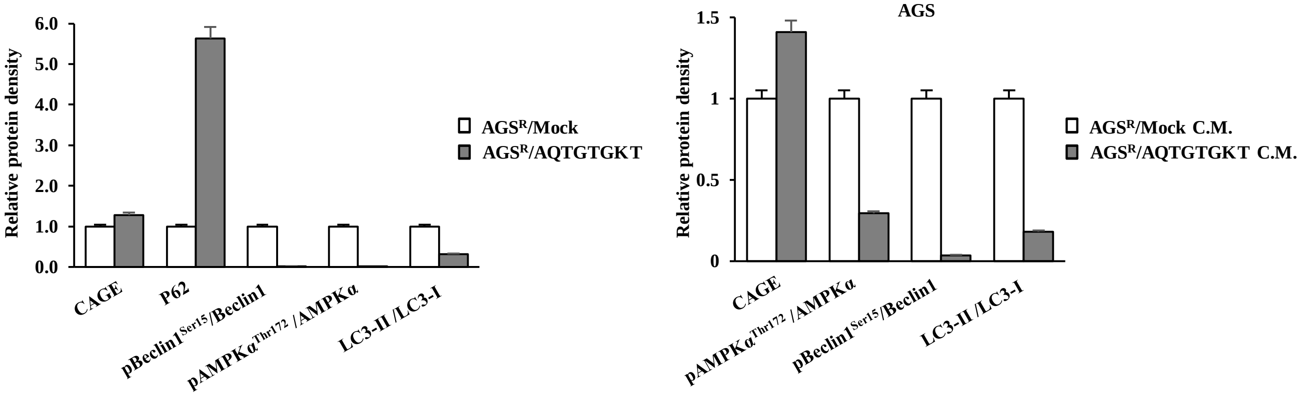

Figure S5

A

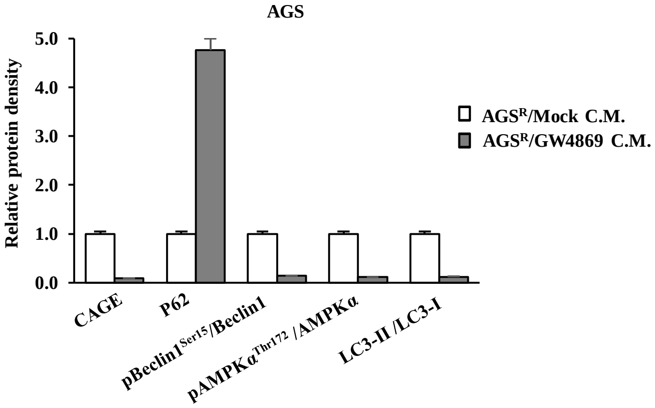

Figure S6

A

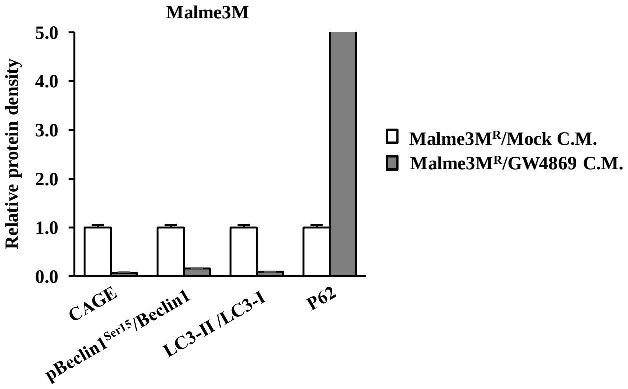

B

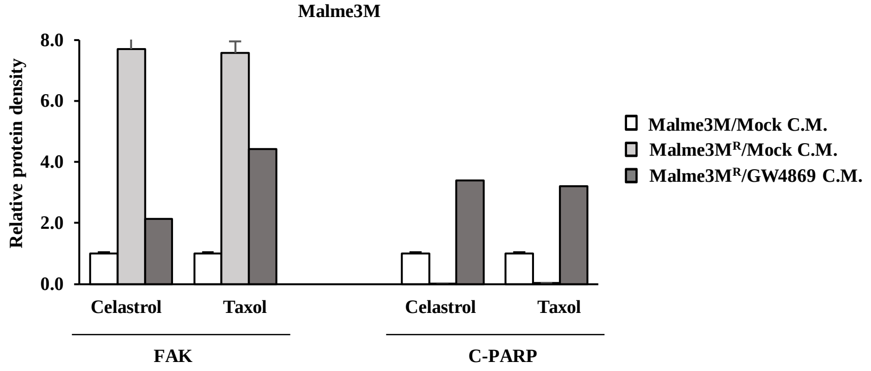

D

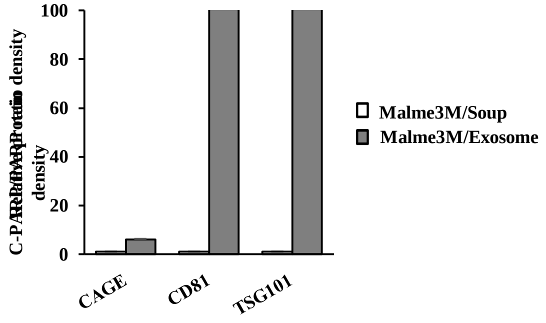

Figure S7

A

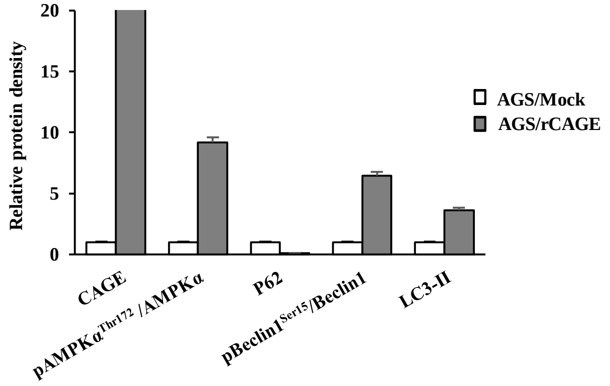

D

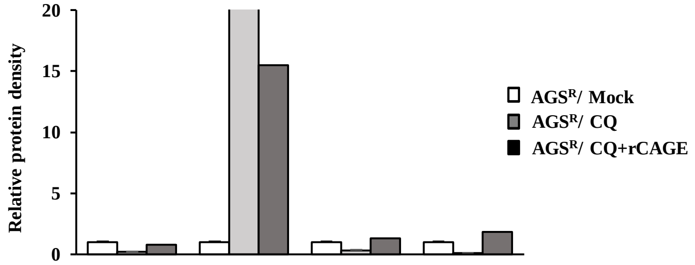

E

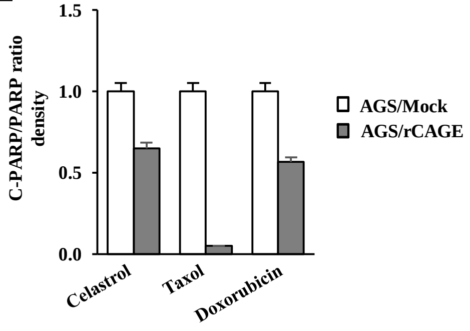

Supplement: Supplementary file 1 [file Data_Sheet_1.PDF]
