# Supplementary material for: The CAGE–MiR-181b-5p–S1PR1 Axis Regulates Anticancer Drug Resistance and Autophagy in Gastric Cancer Cells
Source: Front Cell Dev Biol. 2021 May 25;9:666387. doi: 10.3389/fcell.2021.666387 (PMC8185229; doi:10.3389/fcell.2021.666387)
Supplement: Supplementary file 2 [file Data_Sheet_2.docx]

**Supplementary figure legend**

**Figure S1.** Anti-cancer drug resistant melanoma cells display enhanced autophagic processes. **(A)** Immunoblot and immunoprecipitation were performed. Representative blots of three independent experiments are shown. **(B)** After 48 hours of transfection with the indicated construct, immunoprecipitation was performed. **(C)** Malme3M^R^ cells display the enhanced autophagic process in comparison with Malme3M cells. The black arrows indicate autolysosomes. The white arrows indicate multivesicular bodies.

**Figure S2.** Knockout of CAGE decreases autophagic flux, invasion potential, growth rate, and tumor spheroid forming potential. **(A)** Immunoblot was performed. Representative blots of three independent experiments are shown. **(B)** LC puncta expression was determined. ***, *p<0.001*. **(C)** Invasion potential of each cancer cell line was determined (left). Average values of three independent experiments are shown. Immunoblot was performed (right). ***, *p<0.001*. Representative blots of three independent experiments are shown. **(D)** IC_50_ values was determined by MTT assays. Each cell line was treated with the indicated anti-cancer drug for 48 hours. **(E)** The tumor spheroid forming potential was determined. *, *p<0.05*; **, *p<0.01*. **(F)** Colony forming potential was determined. *, *p<0.05*; **, *p<0.01*.

**Figure S3.** CAGE increases autophagic flux, invasion potential, and confers resistance to anti-cancer drugs. **(A)** CAGE CRISPR-Cas9 cell line was transfected with the indicated construct. After 48 hours after transfection, immunoblot was performed. Representative blots of three independent experiments are shown. **(B)** After 48 hours of transfection with the indicated construct, immunoblot (left) and invasion assays (right) were performed. ***, *p<0.001*. Representative blots of three independent experiments are shown. **(C)** Same as **(B)** except that colony formation assays were performed. **(D)** Same as **(B)** except that tumor spheroid forming potential was determined. **(E)** After 24 hours of transfection with the indicated construct, cells were then treated with celastrol (1 μM) or taxol (1 μM) for 24 h. Representative blots of three independent experiments are shown.

**Figure S4.** CAGE-binding peptide regulates autophagic flux. **(A)** AGS^R^ cells were transfected with the indicated peptide (10 μM) for 24 hours. Representative blots of three independent experiments are shown.  **(B)** AGS^R^ cells were transfected with the AQTGTGKT peptide with or without biotin label. At 48 hours after transfection, immunoprecipitation was performed. **(C)** Same as **(A)** except that the number of LC3 puncta was determined. **, *p<0.01*. **(D)** AGS^R^ cells were transfected with the indicated peptide (10 μM) for 24 hours. Culture medium was then added to AGS cells for 24 hours, followed by immunoblot. Representative blots of three independent experiments are shown.

**Figure S5.** Inhibition of exosomes formation regulates autophagic flux. **(A)** AGS^R^ cells were treated with or without GW4869 (20 μM) for 24 hours. Culture medium was then added to AGS cells for 24 hours. Representative blots of three independent experiments are shown. **(B)** Same as **(A)** except that the number of LC3 puncta was determined. ***, *p<0.001*.

**Figure S6.** Soluble factors regulate anti-cancer drug resistance and autophagic flux. **(A)** Malme3M^R^ cells were pretreated with or without GW4869 (20 μM) for 24 hours. Cells were then treated with the indicated anti-cancer drug for 24 hours. Culture medium of Malme3M^R^ cells was then added to Malme3M cells for 24 hours. Representative blots of three independent experiments are shown. **(B)** Malme3M^R^ cells were pretreated with or without GW4869 (20 μM) for 24 hours. Culture medium was added to Malme3M cells for 24 hours. Representative blots of three independent experiments are shown. **(C)** Black arrows in field indicate isolated exosomes (upper panel) and immuno-gold labeling with an anti-CAGE antibody shows the localization of CAGE in the lumen of the exosome (lower panel). **(D)** Immunoblot shows the presence of CAGE in the exosomes of Malme3M^R^ cells.

**Figure S7.** Recombinant CAGE protein regulates autophagic flux and anti-cancer drug resistance. **(A)** AGS cells were treated with or without human recombinant CAGE protein (1 μg/ml) for 24 hours. Representative blots of three independent experiments are shown. **(B)** Same as **(A)** except that invasion assays were performed. **, *p<0.01*. Average values of three independent experiments are shown. **(C)** Same as **(A)** except that the number of LC3 puncta was determined. ***, *p<0.001*. **(D)** AGS^R^ cells were treated with or without human recombinant CAGE protein (1 μg/ml) for 24 hours, followed by treatment with CQ (100 μM) for 24 hours. Representative blots of three independent experiments are shown. **(E)** Same as **(D)** except that cells were treated with or without the indicated anti-cancer drug (each at 1 μM) for 24 hours. Representative blots of three independent experiments are shown.

**Figure S8.** S1PR1 is correlated with tumorigenesis and poor prognosis. **(A)** GEPIA was used for the analysis of S1PR1 expression. **(B)** Images of the IHC analyses of S1PR1 using tissue microarray (n= 40 normal adjacent gastric tissue, n= 40 gastric cancer tissue). Scale bars are shown. **(C)** Box plots of S1PR1 expression determined by the IHC scoring using tissue microarray. Image J software was used to determine S1PR1 expression levels. **, p<0.05*. **(D)** The disease-free and overall survival rates of the patients with gastric cancers were computed with the web tool.
