# Supplementary material for: The CAGE–MiR-181b-5p–S1PR1 Axis Regulates Anticancer Drug Resistance and Autophagy in Gastric Cancer Cells
Source: Front Cell Dev Biol. 2021 May 25;9:666387. doi: 10.3389/fcell.2021.666387 (PMC8185229; doi:10.3389/fcell.2021.666387)
Supplement: Supplementary file 3 [file Data_Sheet_3.docx]

**Supplementary Table 1.** The sequences of microRNA inhibitors and mimics

| **Name** | **Sequence (5'-3')** |
| --- | --- |
| **Inhibitors** | |
| Negative control | CAGUACUUUUGUGUAGUACAA |
| miR-302b-5p | ACUUUAACAUGGAAGUGCUUUC |
| miR-181b-5p | AACAUUCAUUGCUGUCGGUGGGU |
| **Mimics** | |
| Negative control | UUGUACUACACA AAAGUACUG |
| miR-302b | UAAGUGCUUCCAUGUUUUAGUAG |
| miR-181b | AACAUUCAUUGCUGUCGGUGGGU |

**Supplementary Table 2.** The sequences of SiRNAs

| **Name** | **Sequence (5'-3')** | |
| --- | --- | --- |
| Negative control | sense | UUCUCCGAACGUGUCACGUTT |
|  | antisense | ACGUGACACGUUCGGAGAATT |
| SiCAGE | sense | CAGUGAACAGAGUGAUCAA |
|  | antisense | UUGAUCACUCUGUUCACUG |
| SiPAI-1 | sense | CUCUUCAGAGCGGAGUACU |
|  | antisense | AGUACUCCGCUCUGAAGAG |
| SiS1PR1 | sense | CACACAAAAGGUAUGAUCA |
|  | antisense | UGAUCAUACCUUUUGUGUG |

**Supplementary Table 3.** Primer sequences for qRT-PCR

| **Name** | **Sequence (5'-3')** |
| --- | --- |
| U6 | CGCAAGGATGACACGCAAATTC |
| miR-302b-5p | ACTTTAACATGGAAGTGCTTTC |
| miR-181b-5p | AACATTCATTGCTGTCGGTGGGT |
