# Supplementary figures and images for: The CAGE–MiR-181b-5p–S1PR1 Axis Regulates Anticancer Drug Resistance and Autophagy in Gastric Cancer Cells
Source: Front Cell Dev Biol. 2021 May 25;9:666387. doi: 10.3389/fcell.2021.666387 (PMC8185229; doi:10.3389/fcell.2021.666387)

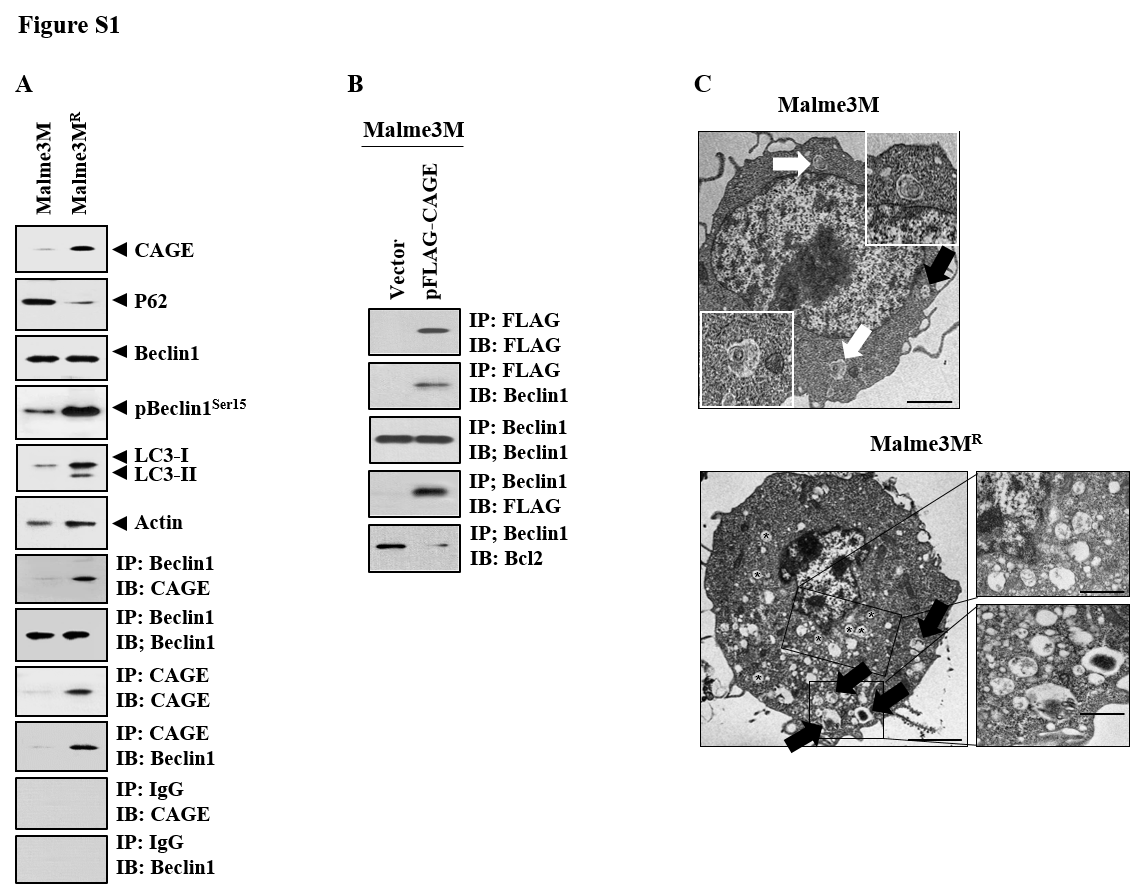

Supplement: Supplementary file 4 [file Image_1.TIF]

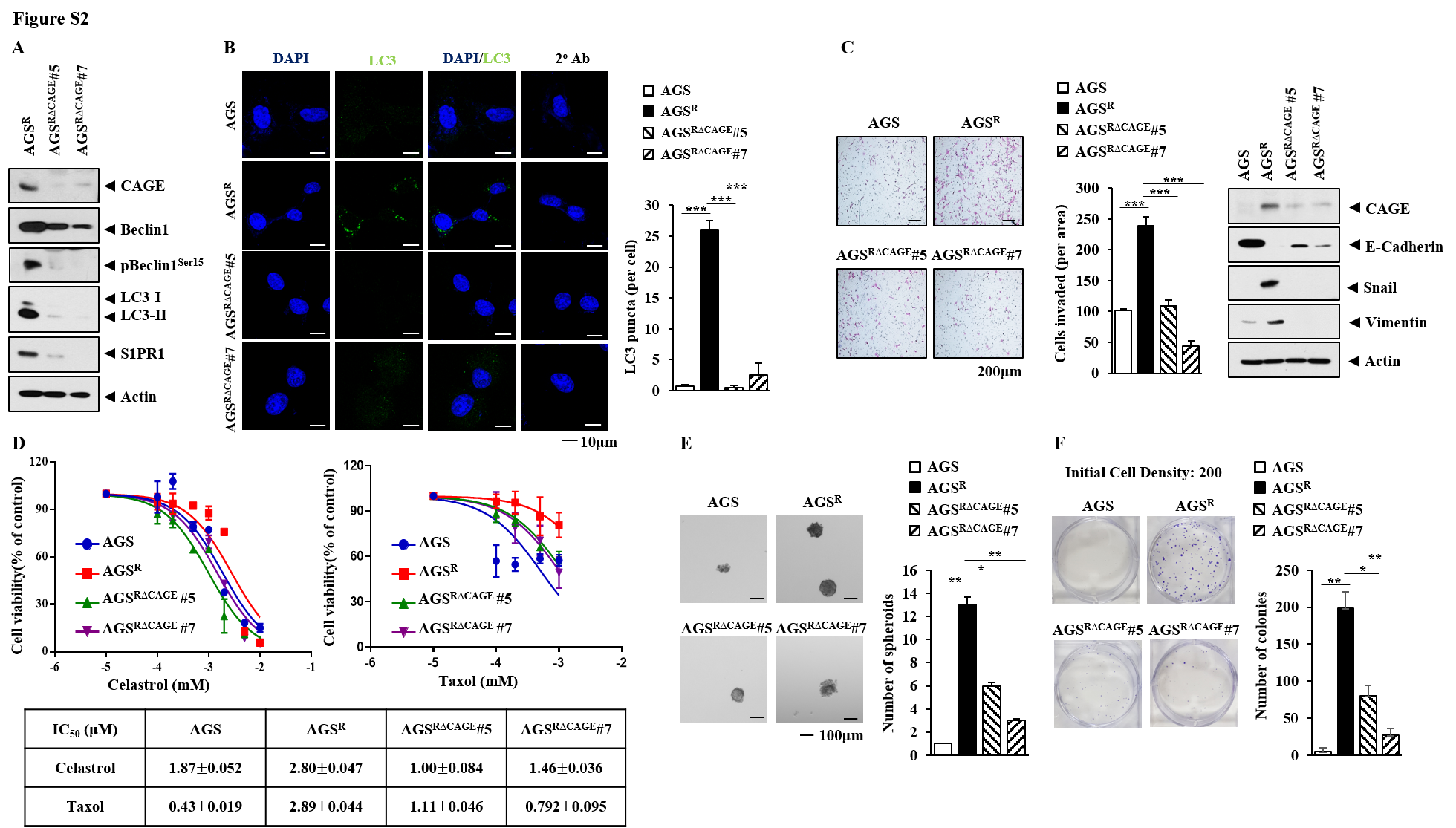

Supplement: Supplementary file 5 [file Image_2.TIF]

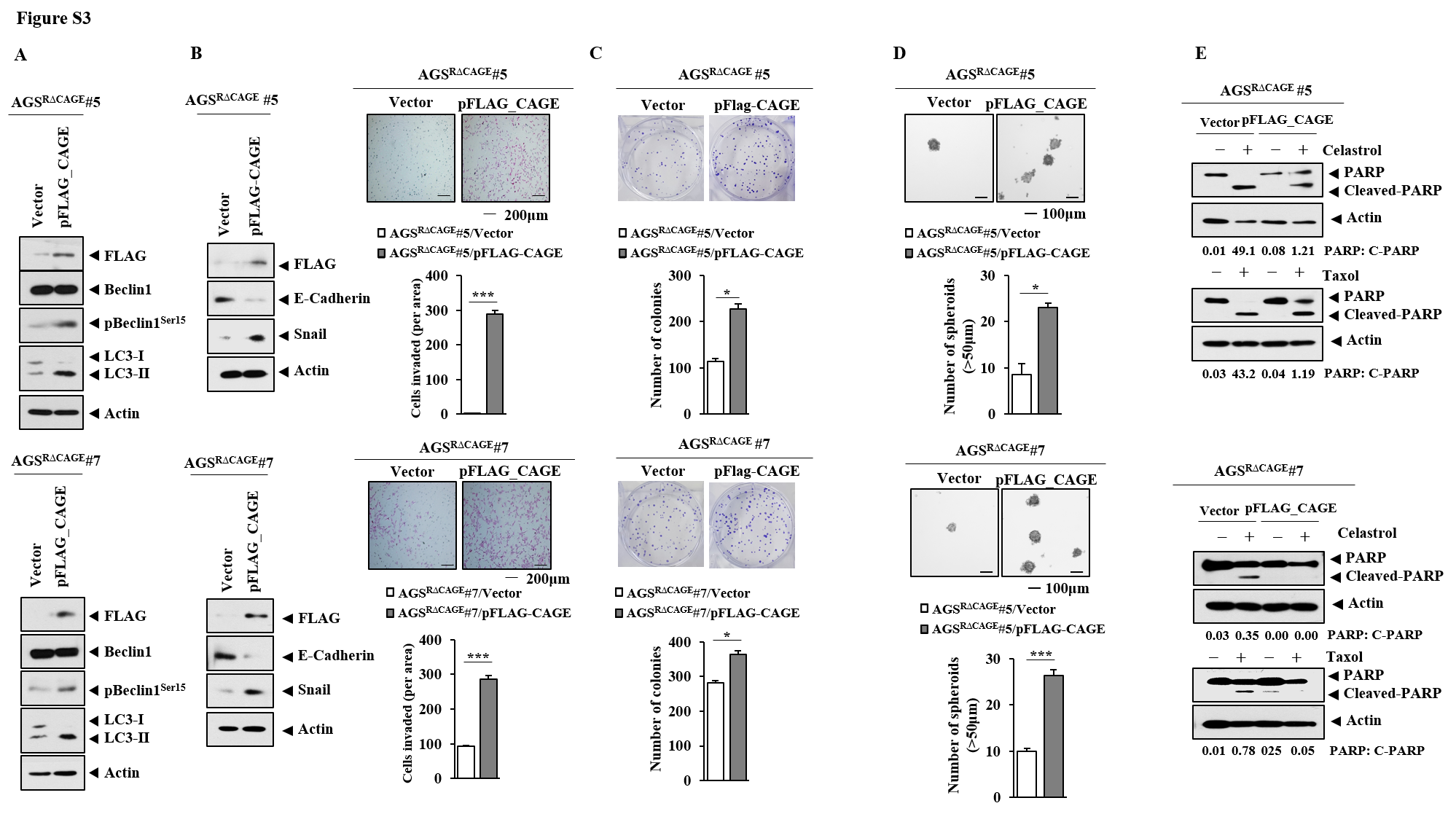

Supplement: Supplementary file 6 [file Image_3.TIF]

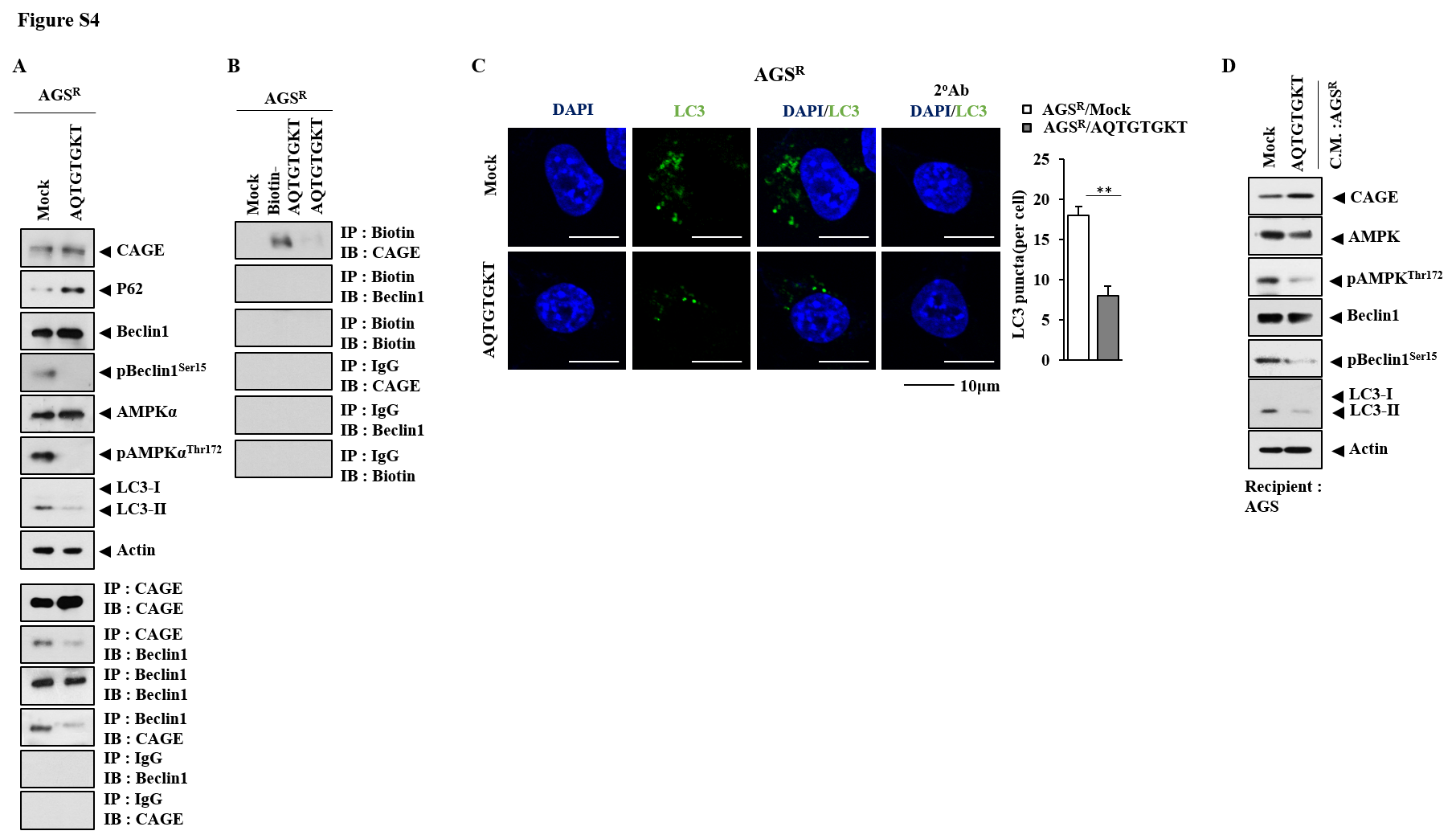

Supplement: Supplementary file 7 [file Image_4.TIF]

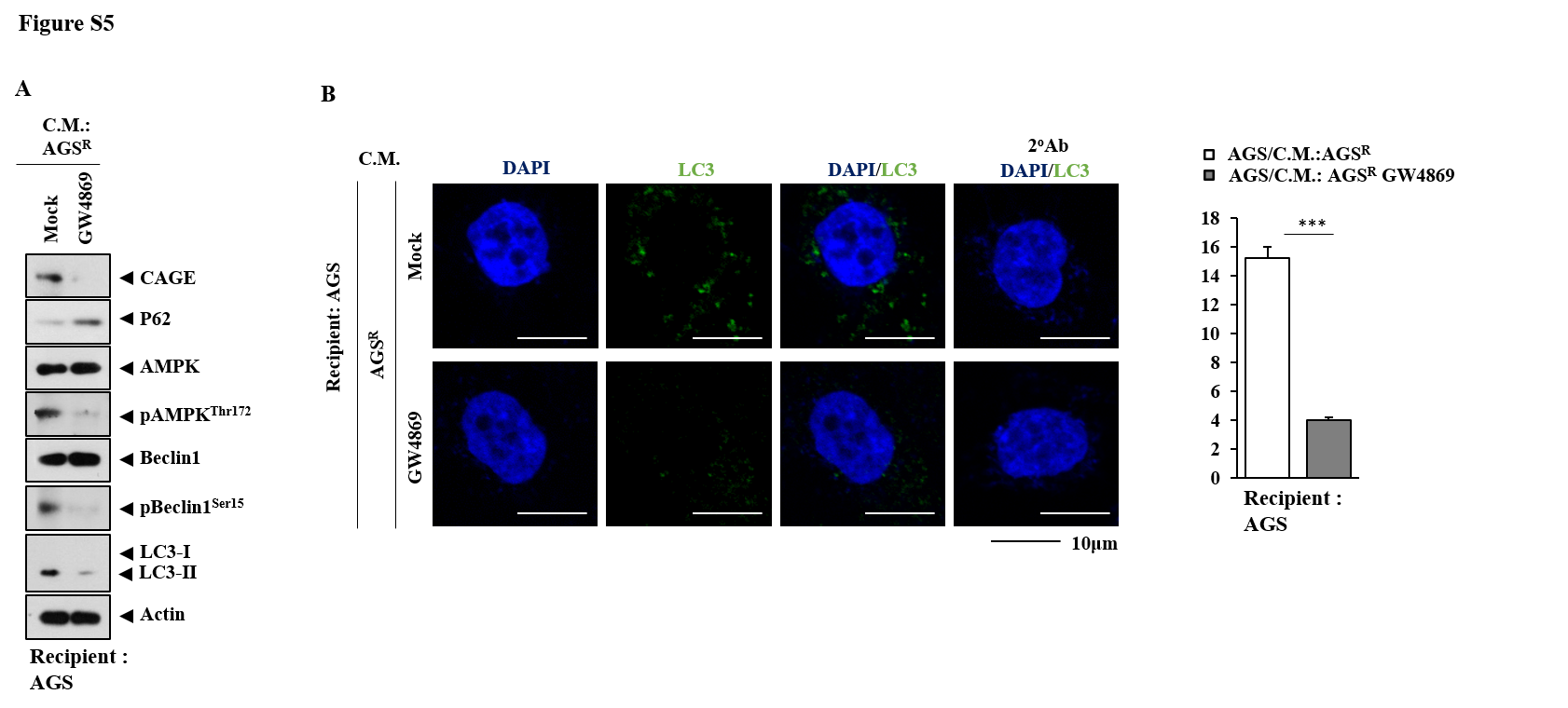

Supplement: Supplementary file 8 [file Image_5.TIF]

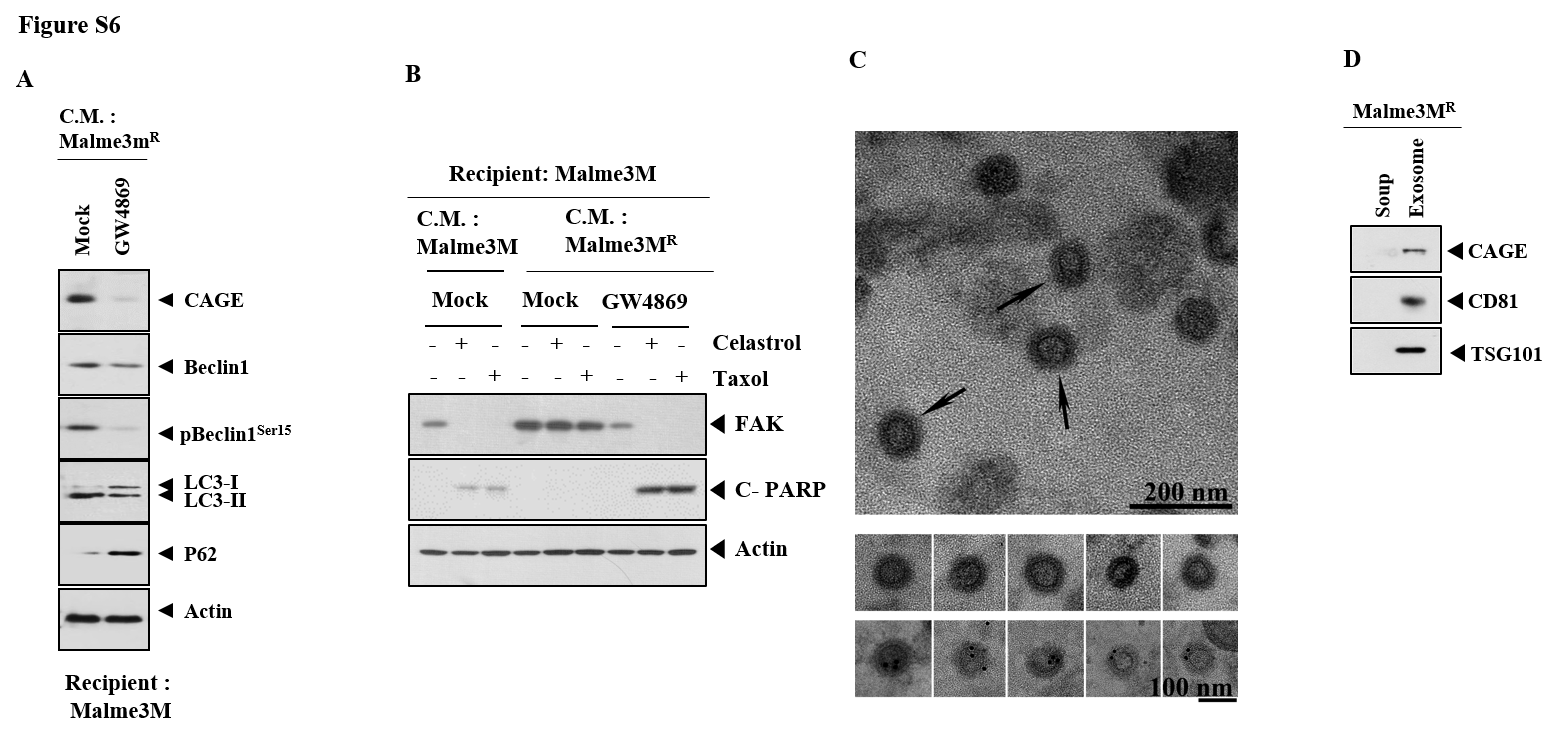

Supplement: Supplementary file 9 [file Image_6.TIF]

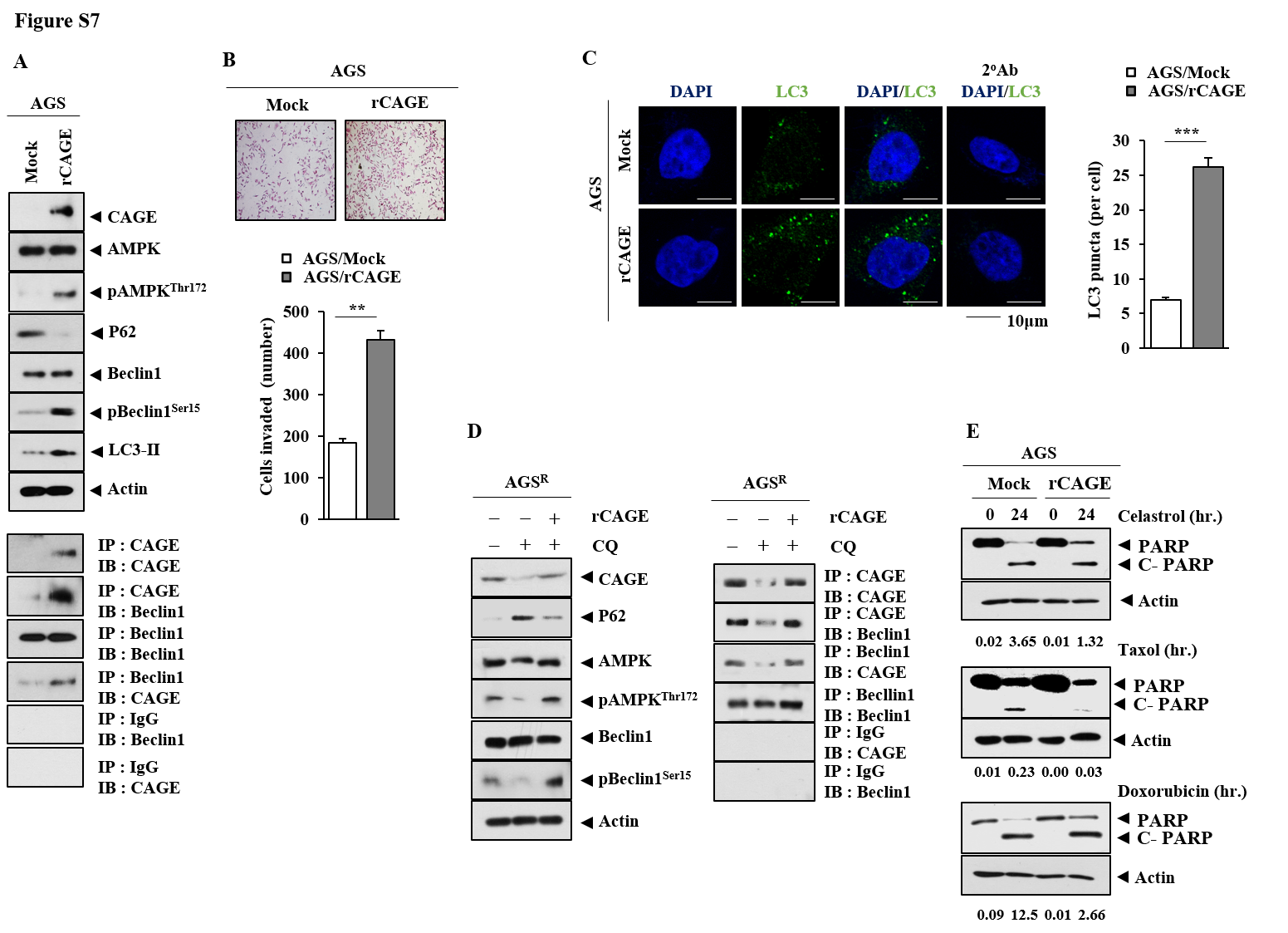

Supplement: Supplementary file 10 [file Image_7.TIF]

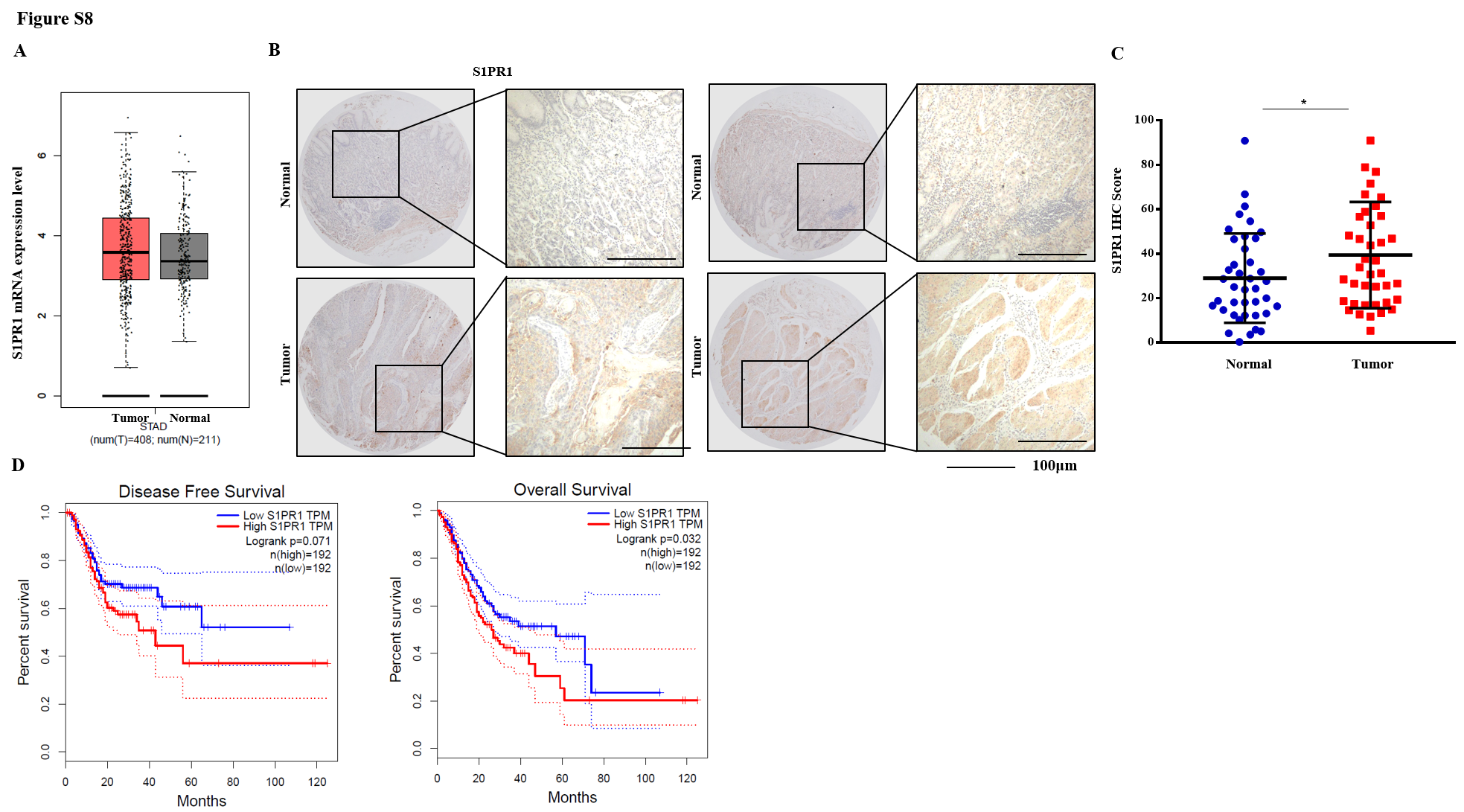

Supplement: Supplementary file 11 [file Image_8.TIF]
